# Comprehending the evolution of gene editing platforms for crop trait improvement

**DOI:** 10.3389/fgene.2022.876987

**Published:** 2022-08-23

**Authors:** Priyanka Dhakate, Deepmala Sehgal, Samantha Vaishnavi, Atika Chandra, Apekshita Singh, Soom Nath Raina, Vijay Rani Rajpal

**Affiliations:** ^1^ National Institute of Plant Genome Research, Aruna Asaf Ali Marg, New Delhi, India; ^2^ International Maize and Wheat Improvement Center (CIMMYT), México-Veracruz, Mexico; ^3^ Department of Botany, Central University of Jammu, Jammu, India; ^4^ Department of Botany, Maitreyi College, University of Delhi, New Delhi, India; ^5^ Amity Institute of Biotechnology, Amity Institute of Biotechnology, Amity University, Noida, India; ^6^ Department of Botany, Hansraj College, University of Delhi, New Delhi, India

**Keywords:** CRISPR/Cas system, base editing, prime editing, epigenome editing, crop improvement

## Abstract

CRISPR (Clustered Regularly Interspaced Short Palindromic Repeats)/Cas (CRISPR-associated) system was initially discovered as an underlying mechanism for conferring adaptive immunity to bacteria and archaea against viruses. Over the past decade, this has been repurposed as a genome-editing tool. Numerous gene editing-based crop improvement technologies involving CRISPR/Cas platforms individually or in combination with next-generation sequencing methods have been developed that have revolutionized plant genome-editing methodologies. Initially, CRISPR/Cas nucleases replaced the earlier used sequence-specific nucleases (SSNs), such as zinc-finger nucleases (ZFNs) and transcription activator-like effector nucleases (TALENs), to address the problem of associated off-targets. The adaptation of this platform led to the development of concepts such as epigenome editing, base editing, and prime editing. Epigenome editing employed epi-effectors to manipulate chromatin structure, while base editing uses base editors to engineer precise changes for trait improvement. Newer technologies such as prime editing have now been developed as a “search-and-replace” tool to engineer all possible single-base changes. Owing to the availability of these, the field of genome editing has evolved rapidly to develop crop plants with improved traits. In this review, we present the evolution of the CRISPR/Cas system into new-age methods of genome engineering across various plant species and the impact they have had on tweaking plant genomes and associated outcomes on crop improvement initiatives.

## 1 Introduction

Over the past decade, the gene-editing platforms have shown tremendous evolution to accommodate the dual concerns of biosafety of edited crops and the efficiency of the platform used. Efficient and rapid genomic sequencing platforms have facilitated a better understanding of plant genomes, particularly when used in conjunction with genome editing (GE). Restructuring genomes *via* introduction of heritable genomic changes for expressing desirable quality traits in crops has been the focus of research for decades. The primitive methods of genome restructuring involved the use of genotoxic agents to introduce random double-stranded breaks (DSB) that were subsequently repaired by inherent non-homologous end joining (NHEJ) pathways resulting in random mutations (Puchta, 2005). After decades of usage of these random mutations generating tools, GE platforms have gone through many phases of improvement over the years. For example, the discovery of sequence-specific nucleases (SSNs) such as zinc-finger nucleases (ZFNs) and transcription activator-like effector nucleases (TALENs) helped to engineer the genome at intended loci by mediating the cleavage of dsDNA. The use of these nucleases induced the native NHEJ pathway for DNA repair (Salomon and Puchta, 1998). This method of GE, however, is both cost- and labor-intensive as it requires the development of sequence-specific nucleases/proteins. In addition, GE using these nucleases was inefficient as unintended off-target edits were introduced by the induction of the error-prone NHEJ repair pathway.

Given the obvious limitations of ZFNs and TALENs, the vacuum was soon filled with the discovery of CRISPR (Clustered Regularly Interspaced Short Palindromic Repeats)/Cas (CRISPR-associated) nucleases. In prokaryotes, the CRISPR/Cas system exists as a means of endogenous small RNA-based adaptive defense mechanism that protects the host bacterial cell *via* sequence-specific recognition and targeted cleavage of viral DNA (Jinek et al., 2012). With an approximate length of 32 bp, the length of CRISPR repeat sequences varies between 21 and 47 bp across prokaryotes. Every CRISPR repeat sequence harbors a unique sequence that is specific to the bacterial species processing it and has, therefore, been conserved over the course of evolution (Karginov and Hanon 2010). CRISPR was first discovered by a Japanese group in 1987 while studying the *iap* gene from the *E. coli* genome (Ishino et al., 1987). They identified CRISPR as homologous repeated sequences of only a few nucleotides interspersed by spacer sequences. Following this, CRISPRs were reported from the archaeal genome, *Haloferax mediterranei* (Mojica et al., 1993). However, the prodigious potential of the CRISPR/Cas9 as a GE platform was discovered just a decade ago (Jinek et al., 2012). To employ this tool, a customized small guide RNA (gRNA) is designed to identify the intended target and guide the associated Cas9 protein to introduce DSBs in the target genomic DNA. Indels are introduced at the target site as the repair pathway *via* NHEJ is triggered. Over the course of evolution of the platform, new variants of Cas proteins have been mobilized to increase the efficiency of the CRISPR/Cas9-mediated GE.

During the past decade, the term “CRISPR/Cas” has evolved into a synonym for GE following which off-targeting instances with the use of CRISPR/Cas systems have reduced manifold (Modrzejewski et al., 2020). However, the goal of achieving “no off-target” remains elusive. In addition, with the involvement of the NHEJ repair pathway, the efficiency of this platform has always been disputable. In the third phase of the evolution of GE platforms, the CRISPR/Cas platform evolved to target the epigenome of an organism which was termed epigenome editing (Konermann et al., 2013). In epigenome editing, chromatin modification at specific genomic loci involves the use of epi-effectors that are comprised of DNA recognition domains (ZFNs, TALENs, or CRISPR/Cas system) and catalytic domains from a chromatin-modifying enzyme. Epigenome editing has been slated to have promising results in numerous basic sciences to decipher functions of chromatin structure and associated modification in phenotypes.

In the fourth phase, the CRISPR/Cas system evolved into a new methodology called base editing, wherein RNA-guided endonucleases were employed to engineer all four possible transitions with increased precision (Komor et al., 2016). One of the major challenges that all of the aforesaid techniques still face is to simultaneously engineer the altered DNA at the intended target sites. These concerns were addressed with the introduction of prime editing, marking the fifth phase in the evolution of GE platforms. Prime editing is largely described as a “search-and-replace” technology that edits the intended genomic loci without generating DSBs (Anzalone et al., 2019). This platform efficiently addresses the concerns of frameshift mutations that arise with the introduction of indels, further reducing off-target mutations. In addition, prime editing can introduce all 12 possible nucleotide substitutions (including transversions and transitions) (Anzalone et al., 2019).

The availability of all new-age GE strategies has not stolen the thunder of the CRISPR/Cas platform owing to the ease of its use and relevance to editing genes in numerous crop plants. However, it is only a matter of time before rapidly changing GE methods will replace present-day CRISPR/Cas systems with more elegant and efficient platforms. With every refinement of the platform, we are getting only closer to generating precise introduced mutations/deletions with reduced off-target effects. In the present review, we evaluate the evolution of GE platforms, such as CRISPR/Cas, epigenome editing, base editing, and prime editing over the last decade to highlight the paradigm shift in our understanding of GE strategies and the relevance of these platforms in present-day agriculture.

## 2 Genome editing using zinc-finger nucleases and transcription activator-like effector nucleases

ZFNs and TALENs represent the first phase of the development of GE platforms. Essentially GE is achieved *via* the introduction of DSBs followed by a homologous repair pathway or the NHEJ-DNA repair pathway. In the first phase of developing GE platforms, SSNs such as ZFNs and TALENs were employed to introduce heritable genomic changes. ZFNs are chimeric enzymes that work as a dimer. Each monomer has 3–5 zinc-finger repeats along with a *FokI* cleavage domain. Each of the zinc fingers is capable of recognizing 3 bp of genomic DNA. Therefore, a ZFN dimer can effectively identify an 18–30 bp DNA with a gap of 5–7 bp (Kim et al., 2007). In plants, the first study involving ZFNs was reported in *Arabidopsis*, wherein heat shock was found to augment ZFN expression. At least 10% of the transgenics obtained displayed the mutations induced by ZFNs in future generations (Lloyd et al., 2005). In maize, ZFNs were employed to introduce a DSB at ipk1, and following this, a herbicide tolerance gene was inserted that resulted in transgenics showing tolerance to herbicide (Shukla et al., 2009). One of the major disadvantages of ZFNs is that the zinc fingers could overlap and are largely dependent on the sequence context around them and the intended DNA segment. Therefore, employing ZFNs becomes both labor- and cost-intensive as for every edit, the zinc-finger array is designed, and the sites available for the edits are limited (Boch and Bonas 2010). Although many studies have reported ZFNs to edit genes, its use as a tool of choice for GE now stands outdated. Another type of nucleases, TALENs, with DNA binding domains, was also employed to engineer genomic changes (Boch and Bonas 2010). Thirty-four tandem repeats are typically present in the DNA binding domain along with repeat-variable di-residue (RVD) comprised of two amino acids at positions 12 and 13, providing the TALENs with the ability to identify the intended target DNA sequence (Cong et al., 2012; Streubel et al., 2012). Like ZFNs, TALENs also introduce DSBs in the intended genomic DNA sequences, completely disrupting the gene and (or) introducing mutations. In comparison to ZFNs, TALENs can be designed for more target sites in the genomic DNA (Boch and Bonas 2010). In rice, TALENs were used to mutate the *OsSWEET* gene to develop transgenic resistance to blight (Li et al., 2012). Similarly, in wheat, transgenic with increased resistance to powdery mildew was developed by employing TALENs induced mutations (Wang et al., 2014). In cabbage, early flowering plants were obtained by employing TALENs (Sun et al., 2013). Like ZFNs, using TALENs is cost- and labor-intensive with limited success, and therefore, their use has now been largely suspended for introducing genomic changes.

## 3 Clustered regularly interspaced short palindromic repeats/Cas system-mediated genetic modification

The CRISPR/Cas systems represent the second phase of evolution in the development of GE platforms. CRISPR/Cas systems are sequence-specific and, therefore, mediate targeted DNA cleavage with increased efficiency. Three major steps are involved in CRISPR/Cas mechanism. The first step is adaptation, wherein a small sequence from the mobile genetic elements (MGEs) is harbored into the host CRISPR resulting in a novel spacer sequence. This adaptive event helps the host bacterial cell evade the attack from the same virus in the future (Barrangou et al., 2007). The selection of the target sequence to be incorporated into the CRISPR array is sequence-specific. In type I, II, and V CRISPR/Cas systems, a small sequence, termed the protospacer adjacent motif (PAM), is found adjacent to the protospacer that is to be incorporated into the CRISPR array. Therefore, PAM is cardinal to both acquiring the protospacer and bringing about the subsequent interference (Datsenko et al., 2012; Zetsche et al., 2015; Fonfara et al., 2016). Although the acquisition mechanism of spacers is not yet fully deciphered, in almost all CRISPR/Cas systems, Cas1 and Cas2 proteins have been found to maneuver the acquisition of the spacer into the CRISPR array (Makarova et al., 2015; Shmakov et al., 2015). Both these proteins are found to be necessary for the acquisition of the spacer (Datsenko et al., 2012). The two proteins form a hetero-hexameric protein complex (Cas1–Cas2), which is central to both excision and incorporation of the protospacer DNA into the CRISPR array (Nuñez et al., 2014). Barring a few exceptions, invariably the spacers are chronologically added to the array (Shmakov et al., 2015). Cas1–Cas2 protein complex is central to protospacer acquisition across most type 1 and type II CRISPR/Cas systems. Therefore, this mode of spacer acquisition stands most well deciphered so far. In the second step, the CRISPR array is transcribed and processed. In addition, the associated *Cas* genes are also transcribed into crRNAs. This step is subtype-specific, and therefore, subtype-specific enzymes are employed. However, broadly across all CRISPR/Cas systems, the CRISPR array is first transcribed into a precursor crRNA (pre-crRNA). Different Cas proteins and ribonucleases cleave and process this in various types of CRISPR/Cas systems to yield a mature crRNA. In the third step, following infection, the mature crRNAs mediate subtype-specific machinery driven mostly *via* Cas proteins to ensure effective cleavage of the MGE. The mechanism of different Cas proteins employed in various CRISPR/Cas systems has been well documented in many studies (Liu L et al., 2020; Talakayala et al., 2022; Wada et al., 2022).

## 4 Classification of the clustered regularly interspaced short palindromic repeats/Cas system

The classification of the CRISPR/Cas systems identified so far is primarily based on the presence of the effector Cas proteins that cleave the invading foreign nucleic acids. The primary classification divides these systems into two classes: Class 1 and Class 2. Class 1 CRISPR/Cas systems employ a multi-protein complex, and Class 2 CRISPR/Cas systems recruit a single effector protein. Further, classification of Class 1 and Class 2 CRISPR/Cas systems into subtypes (I through VI) is dependent on their mechanism of action. The effector module of the CRISPR/Cas system is divided into three stages: the adaptation stage, the expression and processing stage, and the interference stage. In class 1 CRISPR/Cas systems (with types I, III, and IV), type I and type III systems employ a multi-protein complex called the Cascade complex along with Cas3 nuclease-helicase and the Cmr complex for type I, type III-A, and type IIIB CRISPR/Cas systems, respectively (Koonin and Makarova, 2019; Chaudhuri et al., 2022). However, class 2 CRISPR/Cas systems (with types II, V, and VI) employ only one effector protein. In type II and type V CRISPR/Cas systems, the expression and processing of the crRNA are regulated by a single protein such as Cas9 and Cpf1, respectively (Makarova et al., 2015; Amitai and Sorek, 2016). Type VI systems have been recently discovered and are the only CRISPR/Cas systems to target RNA specifically (Chaudhuri et al., 2022). In Class 1 CRISPR Cas systems, type 1 and type III are more prevalent than type IV in diverse bacterial and archaeal populations. However, type II of the Class 2 CRISPR/Cas system is found across all bacterial species (Koonin and Makarova, 2019). Depending on their function, Cas proteins can be primarily classified into four categories; recombinases/nucleases that aid the acquisition of spacers, ribonucleases that regulate the processing of crRNAs, scanning complexes like the crRNP complex, and nucleases that mediate the cleavage of the intended target sequences (Van Der Oost et al., 2014).

Class 1 CRISPR systems, types I and III, bear structural similarities suggesting evolution *via* a common ancestor (Chaudhuri et al., 2022). In addition, they employ Cas9 endonuclease to process crRNA. Type I CRISPR/Cas systems are further divided into six subtypes, types I-A, I-B, I-C, I-D, I-E, and I-F, depending on the distinct PAMs that the subunits require to regulate recognition and acquisition. The type III systems are divided into four subtypes, type III-A, III-B, III-C, and III-D, based on variation in adaptation, recognition, and interference modules of the effector protein complex. Chaudhuri et al. (2022) discussed the further classification of type I and type III into subtypes at length. Class 2 CRISPR/Cas system is divided into three types, types II, V, and VI. Out of these, the type II system is the most dissected and well-understood system so far (Koonin and Makarova, 2019; Chaudhuri et al., 2022). This system employs the Cas9 endonuclease as the effector. Type V system uses a single effector protein, Cas12. However, Cas12 has six subtypes, types V-A, V-B, V-C, V-D, V-E, and V-U, that identify distinct PAM sequences (Chaudhuri et al., 2022). Owing to obvious advantages such as smaller size, no dependency on tracr for target recognition, and asymmetric cleavage sites, Cas12 has now been actively replacing the Cas9 system for GE in many animal and plant species. Type VI systems are characterized by the presence of higher eukaryotes and prokaryotes nucleotide-binding (HEPN) domains with RNase activity (Koonin and Makarova, 2019; Chaudhuri et al., 2022). Cas13a was the first protein identified for type VI CRISPR/Cas systems (Chaudhuri et al., 2022). The evolution of type VI-B, such as Cas13b, is thought to have occurred from transmembrane systems, making them unique from type VI systems into a new subtype type VI-B (Chaudhuri et al., 2022). Type VI systems only target RNAs, thus thought to have lower instances of off-targeting and, in turn, do not harm the host cell much. The extensive diversity of the CRISPR/Cas system, as evident by their classification, reflects the evolution of the CRISPR/Cas-based defense mechanism in both archaea and bacteria. In addition, this diversity of CRISPR/Cas systems presents researchers with varied tools of GE to introduce precise changes with efficacy. Table 1 summarizes the classification of the CRISPR/Cas systems identified so far.

**TABLE 1:** Classification of the identified CRSIPR-Cas systems.

Class	Type	Effector module	Class	Type	Effector module
Class I	I-A	Cas8a2, Csa5	Class II	V-B	Cas12b
Class I	I-B	Cas8b	Class II	V-C	Cas12c
Class I	I-C	Cas8c	Class II	V-D	Cas12d
Class I	I-D	Cas10d	Class II	V-E	Cas12e
Class I	I-E	Cse1, Cse2	Class II	V-F	Cas14
Class I	I-F	Csy1, Csy2, Csy3, Cas6f	Class II	V-G	Cas12g
Class II	II-A	Csn2	Class II	V-H	Cas12h
Class II	II-B	Cas9 (Csx12 subfamily)	Class II	V-I	Cas12i
Class II	II-C	N/A	Class II	V-J	Cas12j
Class I	III-A	Csm2 (small subunit)	Class II	V-K	Cas12k
Class I	III-B	Cmr5 (small subunit)	Class II	VI-A	Cas13a
Class I	IV	DinG (Csf4)	Class II	VI-B	Cas13b, along with proteins, Csx27, and Csx28
Class II	V-A	Cas12a (previously known as Cpf1)	Class II	VI-C	Cas13c
			Class II	VI-D	Cas13d

## 5 Repurposing native clustered regularly interspaced short palindromic repeats/Cas9 for the development of genome-editing platforms

**FIGURE 1:**
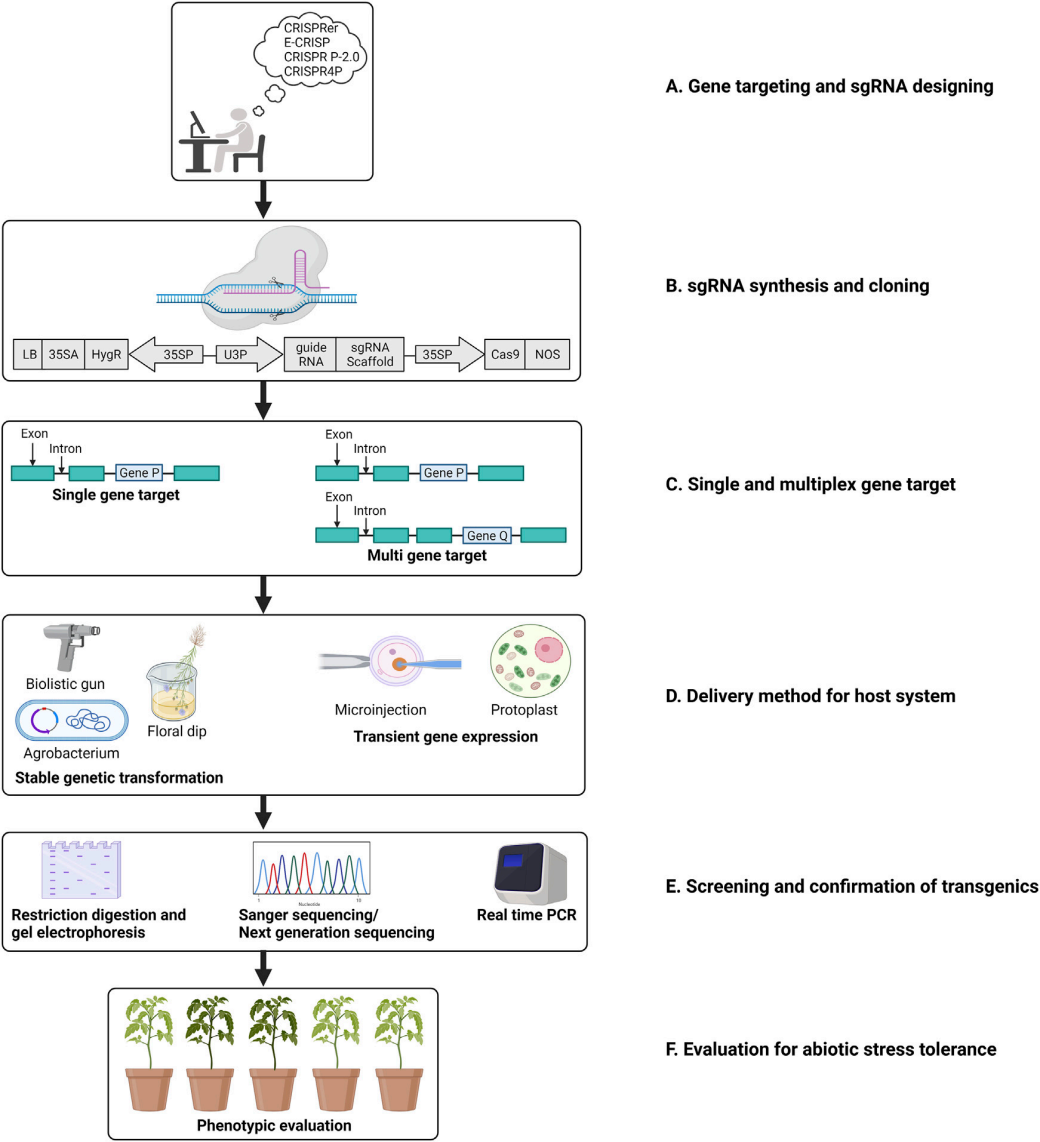
Schematic representation of steps involved in a CRISPR/Cas mediated gene editing in plants. **(A)**. Target gene selection and designing of sgRNA; **(B)**. Engineering the sgRNA in an appropriate binary vector. **(C)**. CRISPR/Cas mediated cleavage via single/multiplex gene editing. **(D)**. Transformation in plants; **(E)**. Screening and evaluation of the crops edited; **(F)**. Evaluation of the plants for selecting transgene-free plant with edited gene(s) regulating the trait of interest (adapted from Jaggannath et al. 2018).

Class II CRISPR/Cas systems were found to be most suitable for development into a tool for genetic manipulation owing to the simplicity of their mechanism of action (Makarova et al., 2015). Type II CRISPR/Cas systems employ Cas9 protein that relies only on an RNA complex of crRNA:tracrRNA that is easy to engineer into a single guide DNA (gDNA) molecule (Jinek et al., 2012). These systems employ only two components: Cas9, a DNA endonuclease, and a customizable gRNA. A single gRNA is sufficient to direct the cleavage of the intended sequences. The gRNA molecules are customized to contain a sequence that Cas9 recognizes and a target sequence that guides the complex to the intended locus (Anders et al., 2014). To identify the intended target site, the Cas9-sgRNA complex scans the targeted DNA for a PAM site, following this 12 bases (seed region) of gRNAs proximal to PAM pair with the intended target sequence (Semenova et al., 2011). Mismatches in the seed region have been found to affect the activity of Cas9 adversely. However, mismatches in the 5’ PAM distal region are well-tolerated without affecting Cas9 nuclease activity (Liu et al., 2016). Catalytic domains of Cas9, HNH, and RuvC invariably result in a DSB in the DNA. Following this, DSB repair is initiated that is mediated either by homology direct repair (HDR) or the NHEJ pathway. The latter does not require a template for DNA repair and hence is error-prone. NHEJ is the active DNA repair mechanism in nature wherein Cas9-induced DSBs are repaired (Moore and Haber 1996). NHEJ can, therefore, lead to small insertions or deletions that could yield a host of mutations (Calvache et al., 2022; Wada et al., 2022). Such mutations are beneficial while knocking out a targeted gene using CRISPR/Cas9 systems. However, being random and unpredictable makes this mode of DNA repair unsuitable for precise editing of intended genes. To this effect, HDR is a more obvious choice of DSB repair mechanism for incorporation of desired sequences following cleavage by Cas9. In plants, GE HDR relies on a DNA template along with the gDNA and Cas9 for a successful DSB repair (Calvache et al., 2022; Wada et al., 2022). In plants, through genetic engineering, many outstanding repairs have been achieved *via* HDR, leading to gene replacement, DNA correction, and targeted knockouts. Figure 1 illustrates a diagrammatic representation of the adaptation to the CRISPR/Cas9 system in plants for gene editing.

## 6 Applications of clustered regularly interspaced short palindromic repeats/Cas9 system as a powerful tool in crop improvement

**FIGURE 2:**
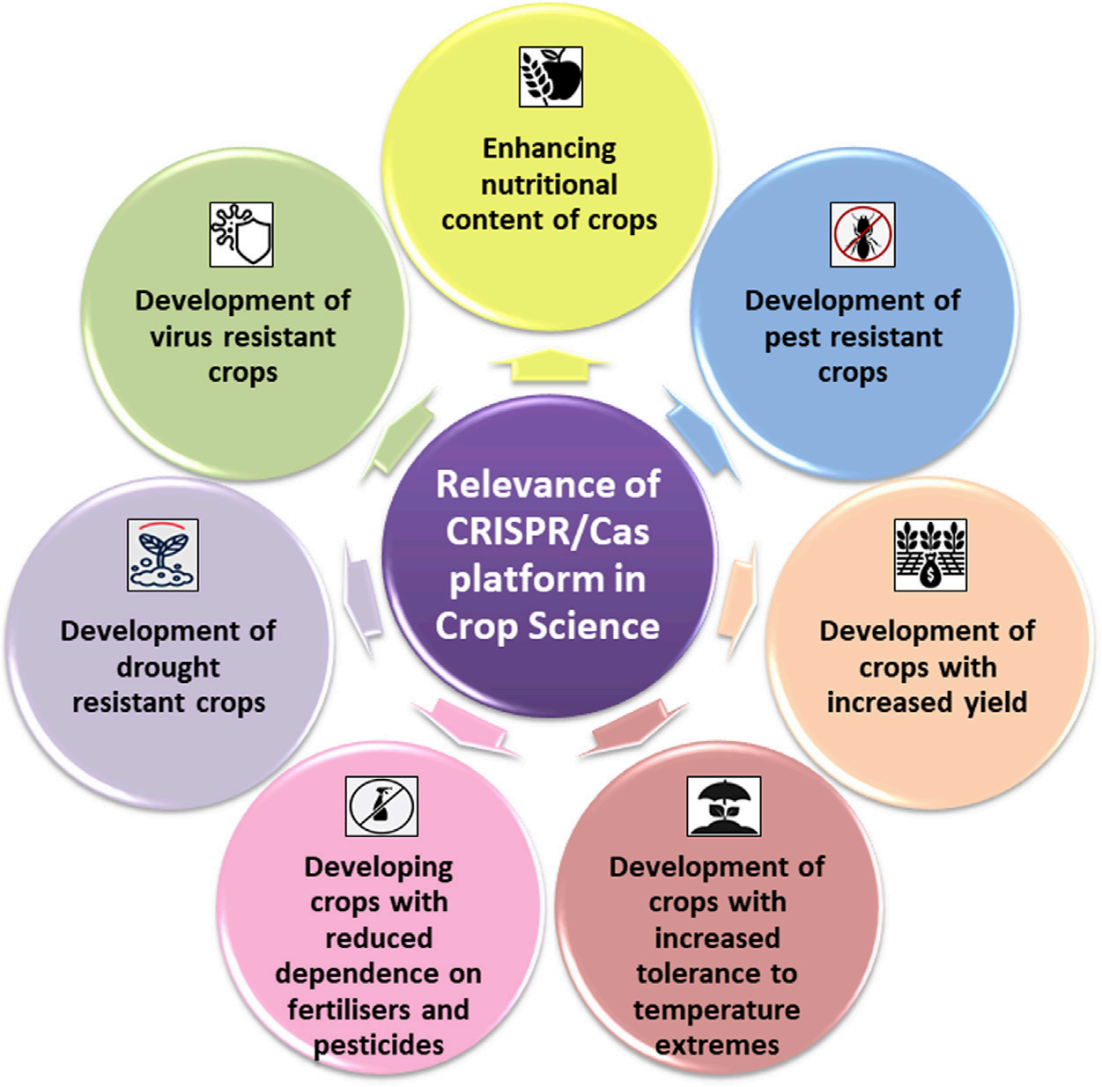
Schematic representations of the domains of crops sciences wherein CRISPR/Cas platforms have largely contributed.

Present-day agriculture faces serious threats from both abiotic and biotic stresses. Rapidly changing climate and exponentially growing world population increase the pressure of ensuring food security for both present and future generations. To mitigate agricultural losses and to aid crops in realizing their full potential, the only sustainable solution is to develop climate-resilient crops. Since its discovery in 2012 as a potential tool for genetic engineering, CRISPR/Cas9 system and its derivatives have rapidly replaced genome engineering methods in crop improvement programs across the globe. In model crops such as maize, a CRISPR/Cas9 mediated knocking and replacement in the *liguleless-1* (*LIG1*) was reported (Svitashev et al., 2016). Similarly, in wheat, CRISPR/Cas9 GE system was employed to introduce targeted mutations in two wheat genes, *TaLox2* and *TaUbiL1*. This study also validated the efficiency of using the CRISPR/Cas9 system in combination with microspore technology in plants for both trait improvement and discovery (Bhowmik et al., 2018). In tomato, complete expression of the susceptibility gene SlyPMR4 was knocked down to generate tomato plants with resistance against powdery mildew (Martínez et al., 2020). CRISPR/Cas9-based GE systems are now employed to improve multigenic traits such as biotic and abiotic stresses in many crops. Table 2 summarizes studies wherein CRISPR/Cas has been used successfully for trait manipulation in crop plants. Figure 2 depicts schematic representation of the domains of crop sciences wherein CRISPR/Cas platforms have largely contributed.

**TABLE 2:** CRISPR/Cas9-mediated improvement in major crop plants.

Plant species	Target gene	Trait of interest	References
Rice (*Oryza sativa*)	*OsAAP6*, *OsAAP10*	Reduced GPC	Wang M et al. (2020)
	*OsBADH2*	Fragrant rice	Kumar et al. (2021)
	*eIF4G*	Resistance to tungro spherical virus	Macovei et al. (2018)
	*OsGAD3*	Increased GABA content	Akama et al. (2020)
	*CrtI*, *PSY*	Increased β-carotene content	Dong et al. (2020)
	*OsGS3*, *OsGW2*, and *OsGn1a*	Increased grain length and width	Zhou et al. (2019)
	*OsDST*	Increased drought and salt tolerance	Kumar et al. (2021)
	*OsPIN5b*, *GS3*, and *OsMYB30*	Increased yield and cold tolerance	Zeng et al. (2020)
	*OsPLDα1*	Low phytic acid content	Khan et al., 2019
Wheat (*Triticum aestivum*)	*TaGW7*	Grain shape	Wang et al. (2019)
	*EDR1*	Resistant to powdery mildew	Zhang et al. (2017)
	*TaGW2*	Grain size	Wang X et al. (2018)
	*α-Gliadin genes*	Low gluten content	Sanchez et al. (2018)
	*TaBAK1-2*, *a-eIF4E*, *Ta-eIF(iso)4E*	Resistance to streak mosaic virus and yellow mosaic virus	Hahn et al., 2021
	*TaSBEIIa*	Grain quality	Li G et al. (2021)
	*TaNP1*	Male sterility	Li et al. (2020b)
Maize (*Zea mays*)	*SH2*, *GBSS*	Super sweet and waxy corn	Dong et al. (2019)
	*Wx1*	Waxy corn	Gao et al. (2020)
	*ZmBADH2a*, *ZmBADH2b*	Aromatic maize	Wang Z et al. (2021)
	*CLE genes*	Enhanced grain yield	Liu et al. (2021)
	*GA20ox3*	Semi-dwarf male plants	Zhang C et al. (2020)
Tomato (*Solanum lycopersicum*)	*ANT1*	Fruit color (purple)	Čermák et al. (2015)
	*CLV3*	Fruit size	Zsögön et al., 2020
	*Psy1*, *CrtR-b2*	Fruit color (yellow)	D’Ambrosio et al. (2018)
	*OVATE*, *Fas*, *Fw2.2*	Fruit size, oval fruit shape	Zsogon et al. (2018)
	*ENO*	Fruit size	Yuste-Lisbona et al. (2020)
	*CRTISO*	Fruit color (tangerine)	Ben Shlush et al. (2021)
	*slyPDS*	Increased lycopene content	Li J et al. (2018)
	*SlNPR1*	Increased drought tolerance	Li et al. (2019)
	*SlCBF1*	Increased cold tolerance	Li R et al. (2018)
	*SlMAPK3*	Increased drought tolerance	Wang et al. (2017)
	*miR482b* and *miR482c*	Resistance to *Phytophthora infestans*	Hong et al. (2021)
	*SlyPMR4*	Resistance against powdery mildew	Martínez et al. (2020)
	*PL*, *PG2a*, *TBG4*	Longer shelf life	Wang et al. (2019)
	*SlLBD40*	Enhanced drought tolerance	Liu et al. (2020)
Rapeseed (*Brassica napus*)	*BnaFAD2*	Improved fatty acid profile	Huang et al. (2020)
	*BnaMAX1*	Improved plant architecture and yield	Zeng et al. (2020)
	*BnaA03.BP*	Compact plant architecture	Fan et al. (2021)

One of the most important applications of CRISPR/Cas9 platforms across the globe has been to engineer disease resistance in crop plants. Plant pathogens such as bacteria, viruses, nematodes, insects, and fungi are the most potent biotic stress factors that impact the yield potential of crops across the globe. Continuously evolving new strains of lethal pests make the battle against the pathogens even more complicated and daunting (Razzaq et al., 2019). Therefore, to protect and aid crops, methodologies routed in concepts of genome engineering have been successfully developed (Jaganathan et al., 2018). Peng et al. (2017) reported the development of varieties of *Citrus sinensis* (Wanjincheng orange) with increased resistance to *Xanthomonas citri*, which is responsible for the citrus canker disease in oranges. In this study, the expression of the gene, *CsLOB1*, which is responsible for the development of the disease, was disrupted using the CRISPR/Cas9 system. Two alleles (*cslob1g* and *cslob1*) exist for the gene CsLOB1. The promoter region of both these alleles inhibits an effector binding site (EBE) that is recognized by the main effector PthA4 of Xcc to drive the expression of *cslob1* and results in the development of the disease. Five independent constructs pCas9/CsLOB1sgRNA were employed to modify the effector binding site EBE in the promoter region of *CsLOB1* alleles. Homologous mutants wherein the EBE was completely disrupted were obtained, displaying no disease development following infection with *Xanthomonas citri* (Peng et al., 2017). In rice, an ethylene-responsive gene OsERF922 was knocked out using the CRISPR/Cas9 tool, which led to a marked reduction in the size and number of the blast lesions. This work led to the development of a rice cultivar with increased resistance against *Magnaporthe oryzae* (Wang et al., 2016). In another study, blight-resistant plants were produced using CRISPR/Cas9 system-mediated targeted mutagenesis of the *SWEET13* gene (Zhou et al., 2015).

Management of diseases in crop plants is dominated by the frequent use of insecticides to curb yield losses. The development of crops resistant to viruses is, therefore, an efficient strategy to yield a stable yet economically viable alternative (Wang W et al., 2021). To this effect, inducing deletions and introducing point mutations in the genes using the CRISPR/Cas9 system is one of the most organic adaptations of the platform. The eukaryotic translation initiation factor genes such as *eIF4E* and *eIF4G* are an absolute requirement for the translation of RNA viruses (Shopan et al., 2020). Therefore, CRISPR/Cas9 technology has been employed in numerous plant species to engineer induced mutations in these genes. In *Arabidopsis*, point mutations in *eIF(iso)4E* gene were found to impart complete resistance against the turnip mosaic virus (Pyott et al., 2016). Likewise, in cucumber, eukaryotic translation initiation factor *eIF(iso)4E* was engineered using the CRISPR/Cas9 system to generate heritable homozygous point mutations that conferred resistance to the mutants against zucchini yellow mosaic virus, papaya ringspot mosaic virus-W, and vein yellowing virus (Chandrasekaran et al., 2016). In *Nicotiana benthamiana*, sgRNA/Cas9-mediated broad-spectrum immunity was achieved against viruses such as beet curly top virus, *Tomato leaf curl Sardinia virus*, *Tomato yellow leaf curl virus*, and *Cotton leaf curl Kokhran virus* (Ali et al., 2016). In rice, the CRISPR/Cas9 system was used to generate *eIF4G* alleles that conferred resistance against the R*ice tungro spherical virus* (Macovei et al., 2018). Recently, Wang et al. (2021) employed the CRISPR/Cas9 system to generate novel *eIF4G* alleles to yield transgenic plants displaying complete resistance to rice black-streaked dwarf virus. Engineering these mutations *via* the traditional backcrossing would have taken years, but using the CRISPR/Cas9 system expedited the process, and the goal was achieved in just a single generation.

The CRISPR/Cas9 system has also been used extensively over the past decade in generating climate-resistant cultivars in various crop species such as cotton, maize, rice, wheat, potato, soybean, and tomato (Khan et al., 2021; Wang et al., 2021; Rahman et al., 2022). In wheat, two regulatory genes (i.e., *TaDREB3* and *TaDREB2*) were mutated using the CRISPR/Cas9 system, which resulted in increased drought tolerance in the mutated plants in comparison to the wild cultivars (Kim et al., 2017). In maize, the *ZmARGOS8* gene that negatively regulates ethylene response was studied using the CRISPR/Cas9 system. The promoter of this gene was knocked out and replaced with maize GOS2 promoter in 5′-UTR of the target gene. The mutant plants were found to overexpress *ARGOS8*, which led to a stupendous increase in the yield in comparison to the wild type under drought conditions during the flowering stage without any yield penalty under irrigated environment (Shi et al., 2017). In rice, the CRISPR/Cas9 system was used to knock out gene *OsRR2*. The homozygous mutants obtained displayed increased tolerance to salinity stress (Zhang et al., 2019). In another study, three genes, *OsPIN5b*, *GS3*, and *OsMYB30*, that determine panicle length, grain size, and cold tolerance, respectively, were simultaneously edited using the CRISPR/Cas9 system (Zeng et al., 2020). T2 generations of the homozygous mutants of these genes displayed increased panicle length, enlarged grain size, and increased cold tolerance, respectively. The CRISPR/Cas9 tool has also been employed for the functional characterization of genes that regulate stress responses in plants. In *Arabidopsis*, three genes (*CBF1*, *CBF2*, and *CBF3*) have been identified to confer cold acclimatization and tolerance. However, the underlying mechanism remained undeciphered owing to the absence of any loss-of-function lines for these genes. Zhao et al. (2016) generated mutants of the cbf gene family, *cbf1*, *cbf2*, and *cbf3*. They generated cbf single, double, and triple mutants using the CRISPR/Cas9 platform. Interestingly, for the three genes, *cbf* triple mutants displayed compromised seedling development and reduced salt tolerance. However, both triple and double (*cbf2cbf3*) mutants displayed increased sensitivity to feeding post-cold acclimatization in comparison to the wild-type control. The *cb1/cb3* double mutants displayed increased resistance, indicating that accumulation of *CBF2* is more important than *CBF1* and *CBF3* in regulating cold acclimation-dependent freezing tolerance. The functional role of many other genes with a potential role in stress tolerance was also investigated in the model system *Arabidopsis*. The expression of *UGT79-B2* and *B3* genes was induced by abiotic stresses such as salinity, drought, and cold. Overexpression of these genes was found to increase the resistance of the transgenics. However, gene *ugt79b2/b3* double mutants generated using the CRISPR/Cas9 system were found to be susceptible to abiotic stresses compared to the wild-type control. The overexpression mutants accumulated anthocyanins, but the *ugt79b2/b3* double mutants that displayed lower levels of anthocyanins were also found to be more susceptible to stresses than the wild-type control plants. These findings also suggested that an array of anthocyanins impart resistance against abiotic stresses (Li et al., 2017). In rice, knockout mutants for the *OsSAPK2* gene were developed for functional characterization of the gene. The mutants showed insensitivity to abscisic acid and increased sensitivity to drought and reactive oxygen species (ROS) during the germination/seedling stage compared to the wild-type control plants. These results suggested the active involvement of the *OsSAPK2* gene in mediating drought tolerance through increased stomatal closure (Lou et al., 2017). In another study, *OsAnn3*, a rice annexin gene, was knocked out in rice using the CRISPR/Cas9 system. The survival ratio of T1 mutant lines was found to be adversely affected, indicating that the expression of *OsAnn3* was central in imparting cold tolerance in rice (Shen et al., 2017).

Drought stress in plants is governed by mitogen-activated protein kinases (MAPKs). In tomato, functional characterization of MAPKs was achieved by knocking down *SlMAPK3* using the CRISPR/Cas9 system (Wang et al., 2017). The resulting *slmapk3* mutants displayed severe wilting symptoms along with lower antioxidant enzymes, increased hydrogen peroxide, and increased membrane damage in comparison to the wild-type control. In another study, a multiplex CRISPR/Cas9 system was used simultaneously to edit five tomato γ-aminobutyric acid (GABA) shunt genes (*CAT9*, *SSADH*, *GABA-TP1*, *TP2*, and *TP3*). These genes are repressors of GABA metabolism. Hence, targeted mutagenesis of these genes led to a 19-fold increase in the accumulation of GABA in fruits and leaves (Li R et al., 2017).

The multiplex CRISPR/Cas9 system has proven to be beneficial in improving yield substantially in various cereal crops. In rice, four genes [i.e., Grain Size 3 (*GS3*), Ideal Plant Architecture 1 (*IPA1*), Grain Number 1a (*Gn1a*), and DENSE AND ERECT PANICLE (*DEP1*)] were edited using the multiplex CRISPR/Cas9 technique. The mutant plants displayed marked improvement in all the aforesaid traits and resulted in better and improved yields concerning tiller number and grain yield (Li et al., 2016). Similarly, multiplex editing using the CRISPR/Cas9 system of four genes, that is, *GS3*, Grain Widths 2, 5, and 6 (*GW2*, *GW5*, and *GW6*), which are negative regulators of grain weight, was investigated in rice. A remarkable improvement was observed in grain weight and size (Xu et al., 2016). The CRISPR/Cas9 system was also employed in rice to knockout three heading date genes (i.e., *Hd2*, *Hd4*, and *Hd5*) (Li et al., 2017). The mutants displayed early heading and higher yield under drought stress conditions. Furthermore, a CRISPR/Cas9 mediated disruption of the *OsSWEET11* gene, known for grain filling and sucrose transportation in rice, led to reduced sucrose concentration and grain weight, which suggested that overexpression of these genes would be beneficial in obtaining a better grain quality (Ma et al., 2017). In wheat, *GASR7* was knocked out using the CRISPR/Cas9 tool, and the resulting mutants showed increased kernel weight (Zhang et al., 2016). In tomato, the use of CRISPR/Cas9 methods has also delivered seedless tomatoes (Ueta et al., 2017). In this study, a novel sgRNA/Cas9 was employed, resulting in additional somatic mutation in *SlIAA9*, a key parthenocarpy gene. The mutation rate was 100%, and there were no off-target mutations. The mutants hence obtained displayed parthenocarpic fruit along with an altered leaf shape.

## 7 Evolution of clustered regularly interspaced short palindromic repeats/Cas9 platform for precise gene manipulation

CRISPR/Cas9 systems have evolved over the years, and many other approaches have also been routed in this technology. As discussed earlier, CRISPR/Cas9-mediated gene editing necessarily introduces DSBs that are subsequently repaired by either NHEJ or HDR mechanisms (Kantor et al., 2020). This results in two major challenges in using CRISPR/Cas9 mechanisms. Firstly, although HDR promises insertion of only sequence-specific DNA, this pathway is synonymous with increased instances of indels and limited efficiency (Song et al., 2017). Secondly, reliance on the HDR mechanism of gene repair restricts gene editing to only dividing cells, adversely affecting the efficiency of this platform in manipulating the disease resistance in plants (Bollen et al., 2018). Many newer technologies that are primarily rooted in the CRISPR/Cas mechanism overcome some of these limitations and are more precise in achieving genome restructuring in plants. Some of these technologies are detailed in the following sections.

### 7.1 Multiplex genome editing

In plants, it is well documented that cellular processes are orchestrated *via* the interplay of several redundant genes. Therefore, editing a single gene from a gene family has not been found to confer the desired phenotype as the redundant genes from the same gene family compensate for the phenotype. In polyploid crop species, this presents an additional layer of complication due to multiple gene dosages or homolog effects. Hence, a more efficient protocol for gene editing is required to aid multiplex gene editing. A single vector system has been used to design many sgRNA cassettes with single or multiple promoters in multiplex gene editing mediated *via* the CRISPR/Cas9 system (Liu et al., 2017). In *Arabidopsis thaliana*, two sgRNAs were successfully employed to disrupt two homologs of *CHLI* (magnesium-chelatase subunit I) to obtain an albino phenotype as both homologs have a function in the photosynthetic mechanism (Mao et al., 2013). In another study in *A. thaliana*, multiplex gene editing was successfully employed to obtain quadruple mutants displaying dwarf phenotype by deploying three gRNAs (Wang et al., 2017).

Further, Čermák et al. (2015) developed a tool kit wherein Csy-type (CRISPR system *yersinia*) ribonuclease 4 (Csy4) was employed along with tRNA-processing enzymes to simultaneously express multiple gRNAs. Using this method, they expressed 12 gRNAs from a single transcript to target deletions in six genes successfully. These Csy4 and tRNA expression systems have been found almost twice as effective in introducing mutations. The use of this platform has been validated in tobacco (*Nicotiana tabacum*), tomato (*Solanum lycopersicum*), wheat (*Triticum aestivum*), barley (*Hordeum vulgare*), and *Medicago truncatula* (Čermák et al., 2015).

Xie et al. (2015) reported an endogenous tRNA-processing mediating gene editing by CRISPR/Cas9 in rice. Soon after, Tang et al. (2016) reportedly employed a single POL II promoter to drive the expression of a hammerhead ribozyme and multiple gRNAs. The ribozyme cleaved distinct sgRNAs, and post-transcription Cas9 processed functional Cas9 and gRNAs. In maize, the CRISPR/Cas9-based gene editing was successfully used to mutate the homologs that determine genic male sterility (Liu et al., 2022). Triple homozygous mutants were obtained that displayed complete male sterility. Over the course of CRISPR/Cas evolution, multiplex gene editing has emerged as an efficient tool to develop “multiple genes-knock-out-cultivars.” Concomitantly, this methodology has enhanced our understanding of gene functions of desired traits that are governed by multiple genes, gene families, or even pleiotropic genes. The technology has also opened vistas for investigating epistatic interactions/associations among genes or gene complexes, especially for complex traits, whose genetic architecture is largely influenced by epistasis.

### 7.2 New Cas variants to broaden the clustered regularly interspaced short palindromic repeats toolbox

Since the discovery of CRISPR/Cas9 mediated gene editing, numerous modifications have been incorporated into this technology to address the issue of incompatible off-target sequences due to gRNA mismatches. There have been many attempts to increase the efficiency of Cas9 enzymes and, at the same time, curb any off-target silencing with the use of enzymes such as dead cas9 (dcas9), SpCas9 Nickase (SpCas9n), and FokICas9 (fCas9) (Cong et al., 2013; Guilinger et al., 2014). Other studies have reported the extraction of Cas9 proteins with increased sequence specificity owing to their novel PAM sequences. Nmecas9 was extracted from *Neisseria meningitidis* specific for PAM sequence 5′-NNNNGATT (Lee et al., 2016). SpCas9 is most commonly used for gene editing with a PAM sequence 5′-NNGRRT (Ran et al., 2015). Modifications have been made for SpCas9 to identify shorter PAM sequences that not only increase the efficiency of the enzyme but also make the delivery of the system easier (Hu et al., 2018). In plants, CRISPR/Cas9 mediated gene editing has been employed in many plant species such as *A. thaliana*, rice, citrus, and tobacco (Jiang and Doudna, 2017). Furthermore, St1Cas9 and St3Cas9 extracted from *Streptococcus thermophilus* have also been employed in CRISPR-mediated gene editing (Jiang and Doudna, 2017). These Cas9 enzymes use different types of tracrRNA and crRNA for identifying PAM sequences (Steinert et al., 2015). Out of all these CRISPR systems employed so far, CRISPR/Cpf1, commonly known as Cas13, is the most popular (Zetsche et al., 2015). Unlike Cas9, Cas13 requires only a sgRNA with 4–5 nucleotide overhangs. In both animals and plants, the Cas13-mediated gene editing has been found to target the desired genes with none or very few off-targets (Endo et al., 2016). Due to their successes, type V CRISPR/Cpf1 has been popular in both plants and animals to engineer gene editing (Zhang et al., 2017). *Francisella novicida*-derived FnCpf1 was used to achieve targeted mutagenesis in both tobacco and rice. Similarly, Lachnospiraceae-derived LbCpf1 has also been used to achieve targeted mutagenesis (Yin et al., 2017).

### 7.3 Epigenome editing

Epigenome editing represents the third phase of plant GE, wherein changes are introduced to engineer the chromatin *via* modification of epigenome at specific sites. It involves targeted, locus-specific, reversible, and heritable alterations of the chromatin structure while bringing in no changes in the nucleotide sequences in the genomes by using epi-effectors. Epi-effectors are the epigenome engineering tools that represent a programmable DNA binding/DNA recognition domain in the genome. Additionally, the catalytic domains of chromatin-modifying enzymes (DNA methyltransferases and histone acetylases) represent components of an Epi-effector. Different epigenome editing tools are available for creating, erasing, and reading various epigenetic codes in plants (Jeltsch and Rots, 2018; Miglani and Singh, 2020; Miglani et al., 2020).

Currently, epigenome editing has been performed through three molecular platforms: zinc-finger proteins (ZFPs), transcription activator-like effectors (TALEs), and CRISPR and dead CRISPR/Cas proteins. These act as DNA-binding domains (DBDs), and after interaction with epigenetic domains, they modify the epigenetic marks at targeted sites in the genome to bring about a restructuring of chromatin architecture and gene expression. The principle of epigenomic editing rests on the formation of fusion proteins between a designed DBD (ZFPs/TALEs/nuclease null or dead Cas9) that targets an attached enzymatic domain (chromatin modifiers; DNA methyltransferases (DNMTs) or histone acetyltransferases (HATs) to define genomic target sites. Hence, the DNA sequences of the target genomic site are presented to DNA-binding protein domains that affect DNA function in the presence of an enzymatic effector domain. This way, epigenome editing allows the precise modification of individual chromatin marks at selected genomic sites (Nakamura et al., 2021).

Besides modulating gene expression, epigenome editing is an appealing approach for understanding the mechanism of chromatin modification, cellular reprogramming, and regulatory functions. It has applications in both basic research involving gene expression studies and application-oriented epigenomic engineering of crop plants. The characterization of epialleles (i.e., alleles that are genetically alike but show variable genetic expression due to epigenomic modifications) is gradually picking up to be fully exploited in future crop improvement programs. Epigenome editing holds great promise in improving crops by creating novel epiallelic diversity that can be exploited for future precision and smart crop epi-breeding (Gahlaut S K et al., 2020; Giudice et al., 2021; Kakoulidou et al., 2021). For epigenome editing, a modified CRISPR/dCas9 known as dead, deactivated, null, or nuclease deficient Cas9 (dCas9) has been created by silencing two mutations of the RuvC1 (D10A) and HNH (H841A) nuclease domains (Qi et al., 2013). The CRISPR-dCas 9 approach is attractive as it helps overcome the limitation of the DBD approach, wherein for targeting a different sequence, a corresponding distinct protein is required, making it difficult to target a wide range of loci in the genomes. In this respect, CRISPR-dCas9 associated system offers flexibility as associated gRNAs help the Cas proteins achieve genomic specificity (Nakamura et al., 2021). A single dCas protein can be reoriented to target different loci simply by altering the sequence of its associated gRNA. This way, the technology offers a flexible platform for targeting almost any genomic sequence (Brocken et al., 2018). Epigenomic editing depends on inducing changes in chromatin architecture to influence gene transcription and relies on primarily inducing reversible and heritable changes in epigenetic marks such as DNA and histones’ methylation, acetylation, and phosphorylation. This results in novel genetic variation in the form of epialleles and has tremendous potential for crop enhancement through epi-breeding. Although several publications have demonstrated the feasibility of epigenome editing in *A. thaliana* (Table 3), its modalities need to be standardized in crop plants for commercial application.

**TABLE 3:** Epigenome editing in the model plant *Arabidopsis thaliana*.

DNB Domain/targeting system/target gene	Epigenome editing/modification	Response	References
ZFN fused to SUVH9	Recruitment of PolV during RdDM through methyl-DNA binding SUVH2 and SUVH9 proteins	DNA methylation and gene silencing	Johnson et al. (2014)
CRISPR dCas9-SunTag based targeting system coupled with tobacco DRM methyltransferase (NtDRMcd)	Manipulation of DNA methylation at FWA promoter	Modification of gene expression, induction of DNA demethylation at FWA, and SUPERMAN promoter affecting gene transcription and triggering a developmental phenotype	Zhong et al. (2014), Papikian et al. (2019)
Mutation of the H3K9 methyl transferase genes *KYP/SUVH4 SUVH5*, *SUVH6*, or the CHG DNA methyl transferase gene *CMT3*	Disruption of histone 3 di-methylation on lysine 9 (H3K9me2) and non-CG DNA methylation *via* mutation of the H3K9 methyl transferase genes *KYP/SUVH4 SUVH5*, *SUVH6*, or the CHG DNA methyl transferase gene *CMT3*	Manipulation of the rate and positions of crossing over (CO). Increase in meiotic recombination in proximity to the centromeres (pericentromeric recombination) and meiotic DNA double-strand breaks (DSBs). Repressive effect of H3K9me2 and non-CG DNA methylation on both meiotic DSB and crossover formation in plant pericentromeric heterochromatin	Underwood et al. (2018)
ZF fusion with catalytic domain human demethylase TET1cd and SunTag-TET1cd system	Demethylation of the promoter of *FWA* (Flowering Wageningen) gene and *CACTA1* transposon	Targeted, complete, highly specific, and heritable demethylation (removal of 5 mC at specific loci in the genome) at *FWA* promoter and activation of gene expression. Reactivation and upregulation of the FWA gene and a heritable late-flowering phenotype. Targeted demethylation and reactivation of heterochromatic TE-CACTA1, although demethylation was incomplete on this locus and remethylation and resilience occurred once the trigger construct was segregated out	Gallego-Bartolomé et al. (2018), Gallego-Bartolomé, (2020)
ZF-RNA directed DNA methylase (RdDM); ZF-MORC6	Co-targeting of both arms of the RdDM pathway, siRNA biogenesis, and co-targeting of Pol IV and Pol V synergistic recruitment	Enhanced targeted *FWA* methylation and silencing, microrchidia- (MORC6-) targeted DNA methylation. Trigger of AGO- and DRM2-dependent methylation	Gallego-Bartolomé et al. (2019), Gallego-Bartolomé, (2020)
CRISPR *dCas9-HAT1* gene	Hyperacetylation at *AREB1* (Abscisic acid-responsive element-binding protein 1) locus resulting in activation of endogenous promoter of *AREB1*	Improved transcription of *AREB1* gene involved in abscisic acid perception. Improved chlorophyll content and drought tolerance due to activation of bZIP TF that can activate several stress tolerance-related genes like RD29A	Paixão et al. (2019)
CRISPR dCas9-TET1	Essential requirement of methylated CG (mCG) and mCHG (where H can be A, C, or T) for targeting RdDM machinery to remethylable loci. RdDm target loci to form stable epialleles in the presence of specific histone and DNA methylation marks	Induction of alternation between two epi-allelic states at a specific locus	Li C et al. (2020)
CRISPR-bacterial methyltransferase MQ1v and CRISPR-SunTagMQ1v Systems	*De novo* induction of CG methylation at different loci with varying efficiency with CRISPR-MQ1v and CRISPR-SunTagMQ1v systems. CRISPR-SunTagMQ1v has shown to be more potent than CRISPR-MQ1v. Development of a CRISPR-based CG-specific targeted DNA methylation system	Improved heritability of induced target-specific CG methylation and high specificity of CRISPR-based MQ1v systems	Ghoshal et al. (2021)

The first successful instance of epigenome editing was achieved in the model plant species *A. thaliana* (Johnson et al., 2014). A ZFN fused to RdDM (RNA-directed DNA methylase) component SU(VAR)3-9 HOMOLOG 9 (SUVH9) was involved in the recruitment of PolV during RdDM mediated *via* methyl-DNA binding SUVH2 and SUVH9 proteins at the FWA target to display DNA methylation induced gene silencing. Many other components of RdDM, such as SHH1, NRPD1, RDR2, DMS3, and RDM, when joined with ZFs, have also been shown to induce methylation at the FWA target in *A. thaliana* (Gallego-Bartolomé et al., 2019). A CRISPR dCas9-SunTag-based targeting system coupled with tobacco DRM methyltransferase (NtDRMcd) was used to target DNA methylation in *A. thaliana* (Zhong et al., 2014; Papikian et al., 2019). It resulted in the induction of DNA demethylation at FWA and SUPERMAN promoters affecting gene transcription and triggering a developmental phenotype. Further, a repressive effect of H3K9me2 and non-CG DNA methylation on both meiotic DSB and crossover formation in plant pericentromeric heterochromatin resulted in manipulation of the rate and positions of crossing over. Increase in meiotic recombination in proximity to the centromeres (pericentromeric recombination) and meiotic DNA double-strand breaks (DSBs) in Thale Cress (Papikian et al., 2019). Recently, Gallego-Bartolomé et al. (2018), Gallego-Bartolomé et al. (2019), and Gallego-Bartolomé (2020) used ZF and CRISPR-dcas9-SunTag systems fused with the catalytic domain of human demethylase TET1cd to test several RdDM components such as RNA-dependent RNA polymerase 2 (RDR2), Microchidia 1 and 6 (MORC1 and MORC6), RNA directed methylation 1 (RDM1), and defective in meristem silencing 3 (DMS3) to induce targeted DNA methylation/demethylation at *FWA* locus in *A. thaliana*. ZF fusion with catalytic domain human demethylase TET1cd and SunTag-TET1cd system resulted in demethylation of the promoter of *FWA* (Flowering Wageningen) gene and CACTA1 transposon and activation of gene expression. While the fusion of ZF-RdDM and ZF-MORC6 enhanced targeted FWA methylation, Microrchidia (MORC6) targeted DNA methylation and triggered AGO- and DRM2-dependent methylation and gene silencing in *A. thaliana* (Gallego-Bartolomé et al., 2019; Gallego-Bartolomé, 2020). These studies provide important experimental evidence to design and utilize a highly targeted and heritable DNA methylation/demethylation system to modulate gene expression in crop plants.

Fusion of CRISPR *dCas9-HAT1* gene resulted in hyperacetylation at *AREB1* (abscisic acid-responsive element-binding protein 1) locus leading to activation of endogenous promoter of *AREB1*. This improved transcription of the *AREB1* gene involved in ABA perception improved chlorophyll content and drought tolerance due to the activation of bZIP TF, which can activate several stress tolerance-related genes such as *RD29A* (Paixão et al., 2019). Further, Li et al. (2020a) showed essential requirements of methylated CG (mCG) and mCHG by using CRISPR dCas9-TET1 fusion (where H can be A, C, or T) for targeting RdDM machinery to re-methylate loci. RdDm target loci were shown to form stable epialleles in the presence of specific histone and DNA methylation marks to induce alternation between two epiallelic states at a specific locus.

Recently, Ghoshal et al. (2021) used CRISPR-bacterial methyltransferase MQ1v and CRISPR-SunTagMQ1v and developed a CRISPR-based CG-specific targeted DNA methylation system to achieve *de novo* induction of CG methylation at different loci with varying efficiency. CRISPR-SunTagMQ1v was shown to be more potent than CRISPR-MQ1v. These MQ1v-based tools appear to be attractive as they offer flexibility to induce methylation at different levels at different loci and show high specificity attributed to the Q147L mutation. Further, the study also demonstrated that for some loci, CG methylation alone was enough to silence gene expression, and for these loci, CRISPR-MQ1v and CRISPR-SunTagMQ1v systems were likely to be more efficient than the DRM2-based SunTag system developed by Papikian et al. (2019) described above.

The above examples show the potential of epigenome editing technology in modulating gene expression and showing observable changes in the phenotypes by altering the DNA methylation status at various genetic loci in *A. thaliana*. Similar studies need to be extended to crop species for exploiting the advantages of locus-specific modulation of DNA methylation through epigenome editing. The new tier of epigenetic variability generated by epigenome editing has significant potential in bringing about the genetic enhancement of crop species.

Epigenome editing, as discussed here and in many other reviews (Gahlaut V et al., 2020; Giudice et al., 2021; Kakoulidou et al., 2021), offers opportunities for editing epigenetic codes in plant genomes globally or at selected loci to create novel genetic variability. To harness the benefits of epigenomic editing, however, it is important to define the specific epimark(s) linked with specific phenotypes and agronomic traits of interest. In this context, genome-wide mapping of epigenomic marks and epigenetic target identification are among the current thrust research areas. A few genetic elements controlled by DNA methylation and linked to desired plant traits have been identified. For instance, naturally occurring epi-alleles that accumulate high levels of vitamin E in tomatoes are associated with differential methylation of a SINE retrotransposon located in the promoter region of gene *VTE3(1)* (Quadrana et al., 2014). In cotton, the *COL2* epi-allele is associated with DNA methylation changes and affects flowering time (Song et al., 2017). It is important to accumulate epigenomic data in various crop species to help identify the potential candidate editing targets. Information on genome-wide changes in DNA methylation in response to environmental stress has been gathered in crops such as rice (Guo et al., 2019; Rajkumar et al., 2020), wheat (Kumar et al., 2017), soybean (Song et al., 2012), and sesame (Komivi et al., 2018).

### 7.4 Base editing

**FIGURE 3:**
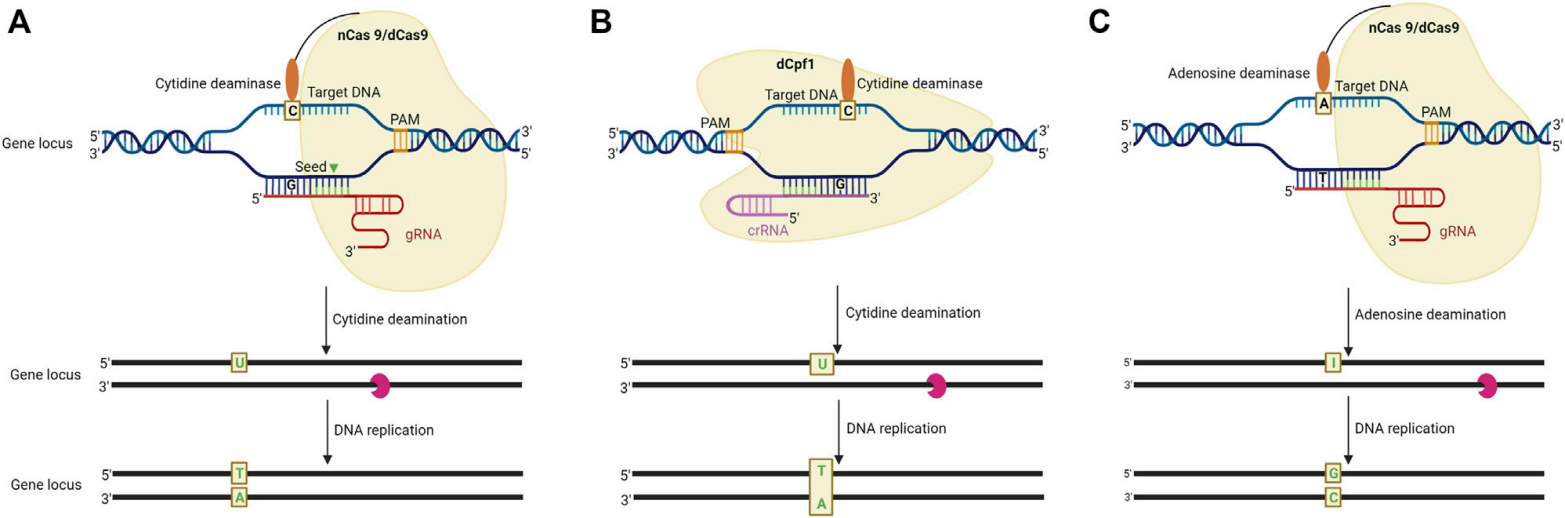
Schematic representation of base editing in plants by using DNA and RNA base editors. **(A)**. CRISPR/Cas9 system-mediated cytosine base editing system (CBE). A sgRNA-dCas9 complex binds to the intended target sequence following this cytidine deaminase catalyses the deamination of cytosine **(C)** resulting in a C-G to T-A conversion. **(B)** CRISPR/Cpf-1 mediated CBE system. In this system, dCpf1 is fused with a cytidine deaminase, to make C-G to T-A conversion in the non-targeted DNA strand. **(C)**. CRISPR/Cas9-mediated adenine base editing system (ABE) employs an Adenosine deaminase and catalytically impaired Cas9 fusion product to bind to the intended target site. The adenosine deaminase catalyses an A (adenine) to I (inosine) change at the target site to introduce A-T to C-C conversion in the DNA strand (adapted from Bharat et al. 2020).

Base editing (BE) is a novel GE technology representing the fourth phase of the evolution of GE platforms wherein a single nucleotide in a DNA or RNA can be substituted irreversibly. The process does not involve a double-stranded breaks (DSB) and hence bypasses the undesirable effects of NHEJ and HDR mechanisms. Of all the previous tinkering tools, BE is the most attractive for the simple reason that here the genome modification is “base-pointed” and precise. It does not involve additions or deletions in the genome (i.e., no change occurs in the DNA content of the organism). Neither does it involve the incorporation of DNA from another organism (i.e., the edited organism does not become a GMO). It minimizes the chances of unintended, unwarranted effects on the phenotype (Rees and Liu, 2018; Deb et al., 2022). With a perfect BE toolbox, one can envisage generating desirable alleles for a trait by simply making the required substitutions. All that is required is a base modifying enzyme linked to a modified endonuclease, such as dCas9, which can target a desired region in the genome but not cause a DSB. Since the advent of this technology in 2016, it has become possible to execute C to T and A to G transition and C to G transversion editing. Figure 3 presents a schematic representation of the working mechanism of the base editing methodology that has been employed for GE.

#### 7.4.1 Cytosine base editors C to T

GE has been revolutionized by engineering the CRISPR/Cas9 to enable cytosine base editing (Komor et al., 2016). The first-generation cytosine base editors (BE1) comprised of catalytically dead dCas9 (D10A, H840A) fused with rat apolipoprotein B mRNA editing enzyme (rAPOBEC1), a cytidine deaminase operating on ssDNA *via* a 16aa XTEN linker at its N-terminus (rAPOBEC1-XTEN-dCas9). Although BE1 was highly efficient in converting C:G to T:A *in vitro*, the same decreased considerably when assessed within cells because of the base excision repair mechanism (BER). To bypass the *in vivo* repair response and overcome decreased efficiency, second-generation cytosine base editors (BE2) were formed by fusion of Uracil DNA glycosylase inhibitor (UGI) to the C-terminal of BE1. This inhibited the action of Uracil DNA glycosylase (UDG), which would otherwise have catalyzed the removal of U, resulting in reversion to C:G through BER. The C:G to T:A conversion efficiency was sought to be further enhanced by generating a nick on the non-edited DNA strand, thereby stimulating the cellular mismatch repair mechanism (MMR), which would replace the G on the nicked strand opposite the U on the target strand by an A, resulting in a U:A, which gets repaired to result in the desired T:A substitution. This resulted in BE3, a BE2 with a dCas9 modified to enable nicking activity (nCas9-H840A), resulting in much more efficient C:G to T:A substitutions (Komor et al., 2016).

#### 7.4.2 Adenine base editors A to G

Although CBEs use naturally occurring cytosine deaminases to convert cytosine to uracil or 5-methylcytosine to thymine, no known adenine deaminases could deaminate the adenosine in DNA. In a significant breakthrough, Gaudelli et al. (2017) used directed evolution to form a modified transfer RNA adenosine deaminase (TadA*), which could catalyze the deamination of deoxyadenosine in an ssDNA resulting in a deoxyinosine. TadA* was joined through the XTEN linked to the N-terminus of Cas9 nickase with a nuclear localization signal (NLS) at its C-terminus (TadA*–XTEN–nCas9–NLS). The group engineered seven generations of ABEs to arrive at ABE7.10, which had high efficiency in converting A:T to G:C (Gaudelli et al., 2017).

#### 7.4.3 Cytosine to Guanosine base editor C to G

It had been observed that although the efficiency of C to T transitions increased considerably by fusing UGI to BE1, in absence of the glycosylase inhibitor, C to T conversions were not so clean and were accompanied by C to G and C to A transversions (Komor et al., 2016). This action of glycosylase, which sought to be inhibited in CBEs for improved recovery of clean C to T substitutions, was tapped for accomplishing C to G transversion in CGBEs. Uracil DNA N-glycosylase (ecUNG) from *Escherichia coli* (Kurt et al., 2021; Zhao et al., 2021) or rat XRCC1 (Chen et al., 2021) were linked to a nCas9 (D10A) and further fused with a rat cytidine deaminase rAPOBEC1 (Chen et al., 2021; Zhao et al., 2021) or its engineered variant rAPOBEC1 (R33A) (Kurt et al., 2021) or with human activation-induced cytidine deaminase (h-AID) (Zhao et al., 2021). The resultant CGBEs or GBEs (glycosylase base editors), UNG-nCas9-APOBEC1, XRCC1-nCas9-APOBEC1, UNG-APOBEC1-nCas9, and h-AID-nCas9-UNG, result in the conversion of C to U and subsequently to G *via* base excision repair (Chen et al., 2021) or by translesion polymerization (Liu et al., 2016). The nicking of the opposite strand triggers the repair machinery of the cell, which converts C:G to G:C.

#### 7.4.4 Dual-base editors

Dual-base editors have recently been developed by merging the cytosine and adenine deaminases in a single editor termed variably as SPACE (synchronous programmable adenine and cytosine editor) (Grunewald et al., 2020), STEMEs (saturated targeted endogenous mutagenesis editors) (Li et al., 2020), ACBE (adenine and cytosine base editor) (Xie et al., 2020), and DuBEs (dual-base editors) (Xu et al., 2021). Grunewald et al. (2020) fused the monomeric TadA of miniABEmax-V82G6 and pmCDA1 of Target-AID5 with the adenine deaminase at the N-terminus and cytosine deaminase at the C-terminus of nCas9 (D10A). Sakata et al. (2020) and Xie et al. (2020) also used the same architecture. Zhang et al. (2020) developed DuBEs (A&C-BEmax) by fusing the two deaminases to the N-terminus and found that hAID-TadA-TadA*linked to nCas9 (D10A) along with two UGIs yielded higher editing efficiency compared to multiplexing with individual deaminase editors in human cells. Li et al. (2020) developed STEMEs by fusing both deaminases, APOBEC3A/ecTadA, to the N-terminus of nCas9 (D10A) and tested them in rice. They reported better C to T and A to G editing with the DuBE than that achieved using co-delivered deaminases and could generate herbicide resistance in rice. Overall, DuBEs were more efficient in C to T edits than A to G. However, the plant DuBE version 1 (pDuBE1) developed by Xu et al. (2021) using TadA-8e and LjCDA1L-4 (*Lethenteron japonicum* CDA1-like 4) fused to the opposite termini of nCas9 (D10A) displayed highly efficient simultaneous A to G/C to T edits (49.7%) in rice calli. Liang et al. (2022) furthered the scope of DuBEs by engineering an AGBE (fusing a CGBE with an ABE), which could render efficient C to G, C to T, C to A, and A to G editing possible in mammalian cells.

#### 7.4.5 Base editing in plants

Base editing (C to T transitions) in plants was demonstrated for the first time in rice (Lu and Zhu, 2017; Ren et al., 2017; Zong et al., 2017; Li et al., 2017). Lu and Zhu (2017) formed a fusion protein, APOBEC1-XTEN-Cas9(D10A), as described by Komor et al. (2016), put it under the ubiquitin maize promoter, and used it for editing *OsNRT1.1B* and *OsSLR1* in rice. Sequencing confirmed C to T (1.4%–11.5%) and C to G (1.6%–3.9%) substitutions in both genes to be more in *SLR1* than *NRT1.1B*. Indels (10%) were much more than the <1% reported by Komor et al. (2016), probably because no uracil glycosylase inhibitor (UGI) was used. Zong et al. (2017) tailored the base editors by including UGI to form pnCas9-PBE (rAPOBEC1-nCas9-D10A-UGI) and pdCas9-PBE (rAPOBEC1-dCas9-UGI) and found that these bring about C to T substitutions in three rice (cell division cycle mutation 48 OsCDC48, nitrate transporter OsNRT1.1B, and a plant architecture gene *OsSPL14*), one wheat (*TaLOX2*), and one maize (*ZmCENH3*) gene with hardly any indels. Cas9 nickase-based editor was more efficient than the one with dCas9. In the same year, Li et al. (2017), while reporting greater than 40% substitutions, proposed that editing efficiency could vary depending on the target locus amongst three targeted loci (one on *OsPDS* and two on *OsSBEIIb*) of rice.

One of the limitations that were obvious in the initial period of the use of this technology was the restriction imposed by the availability or otherwise the canonical PAM sites in a genome. To overcome this challenge, Cas variants/orthologues with relaxed PAM sites both naturally occurring and engineered have been employed. Further, since the first reported use of rAPOBEC cytidine deaminase from a rat in BE1, deaminases sourced from other organisms such as human apolipoprotein B mRNA editing enzyme (hAPOBEC3A) (Gehrke et al., 2018; Wang W et al., 2018), hAID (Hess et al., 2016), *Petromyzon marinus* cytidine deaminase 1 (PmCDA1) (Nishida et al., 2016), and their mutated forms with varying features *vis-a-vis* editing window, size, sequence preference, and so on have been reported (Cheng et al., 2019).

Various proof of concept studies conducted in plants for base editing using natural and engineered variants of Cas in combination with different cytidine/adenine deaminases have been listed in Table 4. A SpCas-9 variant, SpCas9-VQR (D1135V + R1335Q + T1337R), recognizes NGAN and NGNG PAM sites, broadening the reach within a genome (Kleinstiver et al., 2015). Ren et al. (2017) used this variant to develop two CBEs for rice, rBE3 (APOBEC1-XTEN-Cas9n-UGI-NLS) and rBE4 (APOBEC1-XTEN-Cas9nVQR-UGI-NLS), and successfully edited a blast susceptible protein and OsCERK1 (a receptor kinase) with an efficiency of 17%. Steinert et al. (2015) and Kaya et al. (2017) recommended the use of *Staphylococcus aureus* Cas9 (SaCas9) in plants because of its smaller size, longer target sequence, different PAM, and somewhat higher efficiency than spCas9. A variant with three mutations E782K/N968K/R105H (SaCas9-KKH SaKKH) has a relaxed PAM (NNNRRT) compared to the wild type (Kleinstiver et al., 2015). Qin et al. (2019) developed nSaCas9(D10A) and nSaKKH(D10A) nickase-based CBEs (Sa-BE3, SaKKH-BE3, Sa-eBE3, and SaKKH-eBE3) and ABEs (Sa-ABE and SaKKH-ABE/ABE-P5) reporting up to 71.9% cytosine edited (nSaCas9, SLR1 gene) and 63.2% adenine edited (nSaCas9, OsSPL17 gene) rice plants. Veillet et al. (2020) used the nickase SaCas9 (nSaCas9) with PmCDA1 to modify granule-bound starch synthase (StGBSS) and Downy Mildew Resistant 6 (StDMR6) in potato. It recognizes 5’--NNGGAT-3′ as a PAM site and has an editing window from −23 to −22. Nishimasu et al. (2018) engineered spCas9 to recognize NG (spCas9-NG), a relaxed PAM, and used the nickase version fused with activation-induced cytidine deaminase (nSpCas9-NG-AID/Target-AID-NG) to determine their editing efficiencies. Although Target-AID had a better efficiency at the canonical PAM, Target-AID-NG had a wider PAM repertoire and performed better than the former at other PAM sites, whereas xCas9-BE4 (Hu et al., 2018) was the least efficient in mammalian cells. Zhong et al. (2019) tested xCas9(D10A)-rAPOBEC1, xCas9(D10A)-PmCDA1-UGI, and Cas9(D10A)-NG-PmCDA1-UGI in rice and concluded that xCas9(D10A)-based editors were comparable in efficiency to those based on wtCas9(D10A). The former demonstrated better fidelity concerning the protospacer, and Cas9-NG-based editors were more efficient among all three tested at relaxed PAM sequences. Endo et al. (2019) used SpCas9-NGv1 nickase in rice. Veillet et al. (2020) used SpCas9NG-based CBE for editing granule-bound starch synthase (StGBSS) and Downy Mildew Resistant 6 (StDMR6-1) in potato. They also tested the performance of this editor in tomatoes by targeting two PAM sites in the acetolactate synthase (ALS) gene. GGT gave a lower efficiency (32%) than the canonical PAM NGN (64%).

**TABLE 4:** Base editing mediated proof of concept and improvement studies in major crop plants.

Aim	Editor	Plant	Genes targeted	References
Proof of concept/demonstration of editing efficiency	CBE	Rice	*OsNRT1.1B*, *OzSLR1*, *OsCDC48*, *OsSPL14*, *OsSERK1*, *OsSERK2*, *OsPi-ta*, *OsSBEIIb*, *OsPDS*, *OsALS*, *OsAOS1*, *OsJAR1*, *OsJAR2*, *OsCOI2*, *OsSNB*, *OsSPL7*, *OsPMS3*, *OsSPL14*, *OsIPA1-T1, OsMKK6*, *OsEhd1*, *OsPi-d2*, *OsMPK3*, *OsROC*	Lu and Zhu (2017), Zong et al. (2017), Ren et al. (2017), Li P et al. (2017), Ren et al. (2018), Wang et al. (2019), Qin et al. (2019), Sretenovic et al. (2021)
		Wheat	*TaLOX2*	Zong et al. (2017)
		Maize	*ZmCENH3*	Zong et al. (2017)
		*Arabidopsis*	*LFY*	Choi et al. (2021)
		Tomato	*SlALS1*, *SlCYC-B*, *SlDET1*, *SlDDB1*, *SlETR1*, *SlETR2*, *SlHWS*, *SlDELLA*	Hunziker et al. (2020), Kashojiya et al. (2022)
		Rapeseed	*BnaCLV3*, *BnaRGA*, *BnaA3.IAA7*, *BnaDA1*, *BnaALS*	Hu et al. (2020), Cheng et al. (2021)
	ABE	Rice	*OsACC-T1*, *OsALS-T1*, *OsCDC48-T3*, *OsDEP1*, *OsNRT1.1B-T1*, *OsIPA1*, *OsSLR1*, *OsMPK6*, *OsMPK13*, *OsSERK2* and *OsWRKY45*, *OsSPL14*, *OsSPL17*, *OsSPL16*, *OsSPL18*, *OsIDS1*, *OsTOE1*, *OsSNB*, *OsPMS3*, *OsPMS1*, *OsSPL14*, *OsLF1*, *OsIAA13*, *OsSPL7*, *OsSPL4*, *OsMADS5*, *OsWx*, *OsPi*-*d3*, *OsGL2*,* OsGRF3*, *OsSLR1*, *OsWSL5*, *OsZEBRA3* (*Z3*), *OsROC*	Hua et al. (2018), Hua et al. (2019), Wang et al. (2019), Hua et al. (2020a), Sretenovic et al. (2021)
		Wheat	*TaDEP1*, *TaGW2*, *TaALS*, *TaTub*	Li J et al. (2018), Han et al. (2022)
		Tobacco	*NbPDS*	Wang W et al. (2021)
	CGBE	Rice	*OsALS*, *OsCGRS55*	Sretenovic et al. (2021)
		Tomato	*AGO7*	
		Poplar	*PtPDS1*, *PtPDS2*	
	DuBE	Rice	*OsAAT*, *OsACC*, *OsCDC48*, *OsDEP1*, *BADH2-2*, *FSD2-1*, *LAZY1-2*	Li et al. (2020a), Xu R et al. (2021)
Co-editing	CBE	Pear, apple	*PDS*, *ALS*	Malabarba et al. (2021)
Double CBE	CBE	Potato	*StDMR6-1*, *StGBSSI*	Veillet et al. (2020)
Simultaneous base editing	CBE and ABE	Rice	*OsSPL14*, *OsSPL17*, *OsSNB*	Hua et al. (2019)
To introduce premature stop codon		Poplar	*4CL1*, *PII*	Li R et al. (2021)
Resistance to biotic stress	CBE	Rice	*OsPi-d2, OsFLS2*	Ren et al. (2017)
Herbicide tolerance	CBE	Rice, wheat, watermelon, foxtail millet, *Arabidopsis*, potato, pear, tomato, rapeseed	*ALS1*, *ACC*, *GS1*, *TubA2*	Chen et al. (2017), Tian et al. (2018), Zhang A et al. (2019), Veillet et al. (2019), Veillet et al. (2020), Cheng et al. (2021), Kuang et al. (2020), Liu et al. (2020), Wu et al. (2020), Zhang J et al. (2020), Malabarba et al. (2021), Liang Y et al. (2022)
Improved grain/fruit/seed quality	CBE	Rice	Waxy	Li et al. (2020a), Xu et al. (2020), Tra et al. (2021)

Hua et al. (2018) adopted ABE7-10 (Gaudelli et al., 2017), developed adenine base editor plant version 1, ABE-P1 [TadA*7.10-SpCas9(D10A) nickase], and 2, ABE-P2 (TadA*7.10-SaCas9(D10A) nickase), and tested them on two rice genes: ideal plant architecture *OsIPA1* and slender plants *OsSLR1*. In 2019, they made several new versions, ABE-P3, P4, and P5, using SpCas9nVQR (D10A) and SpCas9-VRER (D10A) to increase target genome accessibility. They could successfully edit at four loci: *SPL14*, *SPL17*, *SPL16*, and *SPL18*. With the same set-up, they could demonstrate simultaneous cytosine and adenine editing using ABE-P2 and CBE-P1. Similar to reports in mammalian systems, there were no indels or off-target or any other unplanned base substitutions seen in rice. However, the editing windows were larger in the target genes. Hua et al. (2019) explored the use of SpCas9 and SaCas9 variants for widening the scope of the adenine base editing toolbox. They used nickases of VQR-, VRER-, and SAKKH-SpCas9 engineered variants to form three ABEs, ABE-P3 (pRABEspVQR), ABE-P4 (pRABEsp-VRER), and ABE-P5 (pRABEsa-SaKKH), and two CBEs with spCas9-VRER and saCas9-SAKKH, all of which were designed and tested in rice. The CBE and ABE formed with xCas9 were not efficient. Wang et al. (2021) compared the capabilities of ABE8e and ABE7.10 in *Nicotiana benthamiana* and established that ABE8e (60.87%) was more efficient than ABE7.10 (20.83%).

Sretenovic et al. (2021) studied the applicability of CGBEs, for affecting transversions in plants for the first time. They improvised the three CGBE platforms for successful use in humans (Chen et al., 2021; Zhao et al., 2021; Kurt et al., 2021) for use in three plant species: rice, tomato, and poplar. All three used the rat-derived rAPOBEC1 or its engineered variant rAPOBEC1 (R33A). rAPOBEC1 in combination with ecUNG or rXRCC1 was fused with nCas9 (D10A), whereas rAPOBEC1 (R33A) was linked to rescuing and nCas9 (D10A). Three, four, and two target sites were chosen for editing in rice, tomato, and poplar, respectively. As compared to BE3, all three CGBEs induced better C to G conversions, but the overall efficiency of conversion was less than that reported in humans. The efficiency of editing using SpRY, which is not PAM dependent, was also assessed. The authors achieved C to G editing, although the efficiency varied according to the system and target site. Because this was the first report, much needs to be done to improve the efficiency of plants.

Base editing is still an evolving technology, and many reports primarily demonstrate the successful use of a base-editing toolbox in different plants. This technology can create random variations within genomes, which can be screened and selected for advantageous traits. It also holds a great promise for improvement in traits affected by SNPs. Applications of the technology have been reported mainly as a gain of function for herbicide resistance and disease resistance and improvement in plant architecture, eating, and cooking quality (Table 4).

Base editing of acetyl-CoA carboxylase (*ACC*) and acetolactate synthase (*ALS1*) genes has been shown to confer herbicide resistance in rice (Li et al., 2020b; Liu et al., 2020; Zhang et al., 2020), tomato (Veillet et al., 2019; Veillet et al., 2020), potato (Veillet et al., 2019), watermelon (Tian et al., 2018), apple (Malabarba et al., 2021), pear (Malabarba et al., 2021), oilseed rape (Wu et al., 2020; Cheng et al., 2021), *Arabidopsis* (Chen et al., 2017), foxtail millet (Liang et al., 2022), and wheat (Zhang et al., 2019). The eating and cooking quality (ECQ) is of utmost importance for all cereals, and it is primarily determined by the amylose content in the grain, determined by the *Waxy* *(Wx)* gene-encoded granule-bound starch synthase I (GBSSI) (Li et al., 2016). Xu et al. (2021) used CBEs to develop rice lines expressing a range of amylose content (0%–12%), which improved its ECQ considerably by making several substitutions near the soft rice allele site in Wx. Similarly, Li et al. (2020a) lowered the amylose content in rice grains. Veillet et al. (2020) incorporated base substitutions in the *GBSSI* locus in potato, which could eventually be used for controlling amylose content in the tubers.

Traditional methods of inducing mutations become especially difficult in polyploid species because they possess more than two copies of a gene. Base editing has successfully generated heritable substitutions in polyploid species such as oilseed rape, wheat, and cotton. Hu et al. (2020) used BnA3A1-PBE in rapeseed and demonstrated an editing efficiency of up to 50.5%, much higher than 23.6% reported by Cheng et al. (2021) and 1.8% by Wu et al. (2020). Li et al. (2018) demonstrated slight success (0.1%–1.1%) of *PABE* 1–7 in affecting A to G transitions in the *TaDEP1* and *TaGW2* wheat loci.

It is quite evident that this technology has immense potential, and once the challenges of discovering more efficient, PAM-independent DNA-binding proteins, better deaminases that can affect cleaner edits with zero off-targets, and engineering all possible substitutions are found, base editing can create a revolution in the field of plant sciences in general and crop improvement in particular.

### 7.5 Prime editing

Prime editing marks the fifth phase of evolution in GE platforms. The technique was first developed and standardized in human cells. Prime editing facilitates indels and all 12 possible base-to-base conversions, including transversions and transitions, without triggering the error-prone repair pathways by the DSB (Anzalone et al., 2019). Briefly, in this technique, paired/coupled prime editing guide RNA (pegRNA) is composed of single gRNA that is complementary to the one strand of the targeted DNA along with a primer-binding site (PBS), and the customized sequences to be replaced at the target site fused with Cas9 nickase are also present (Kumar et al., 2021). The PBS region primes to the second DNA strand to drive reverse transcriptase (RT) linked with the Cas9 nickase. RT transcribes and, in the process, copies the information straightaway from pegRNA into the intended target site. Following this, 5′ and 3’ are the single-stranded overhangs integrated into the genomic DNA *via* endogenous DNA repair mechanisms (Anzalone et al., 2019).

**FIGURE 4:**
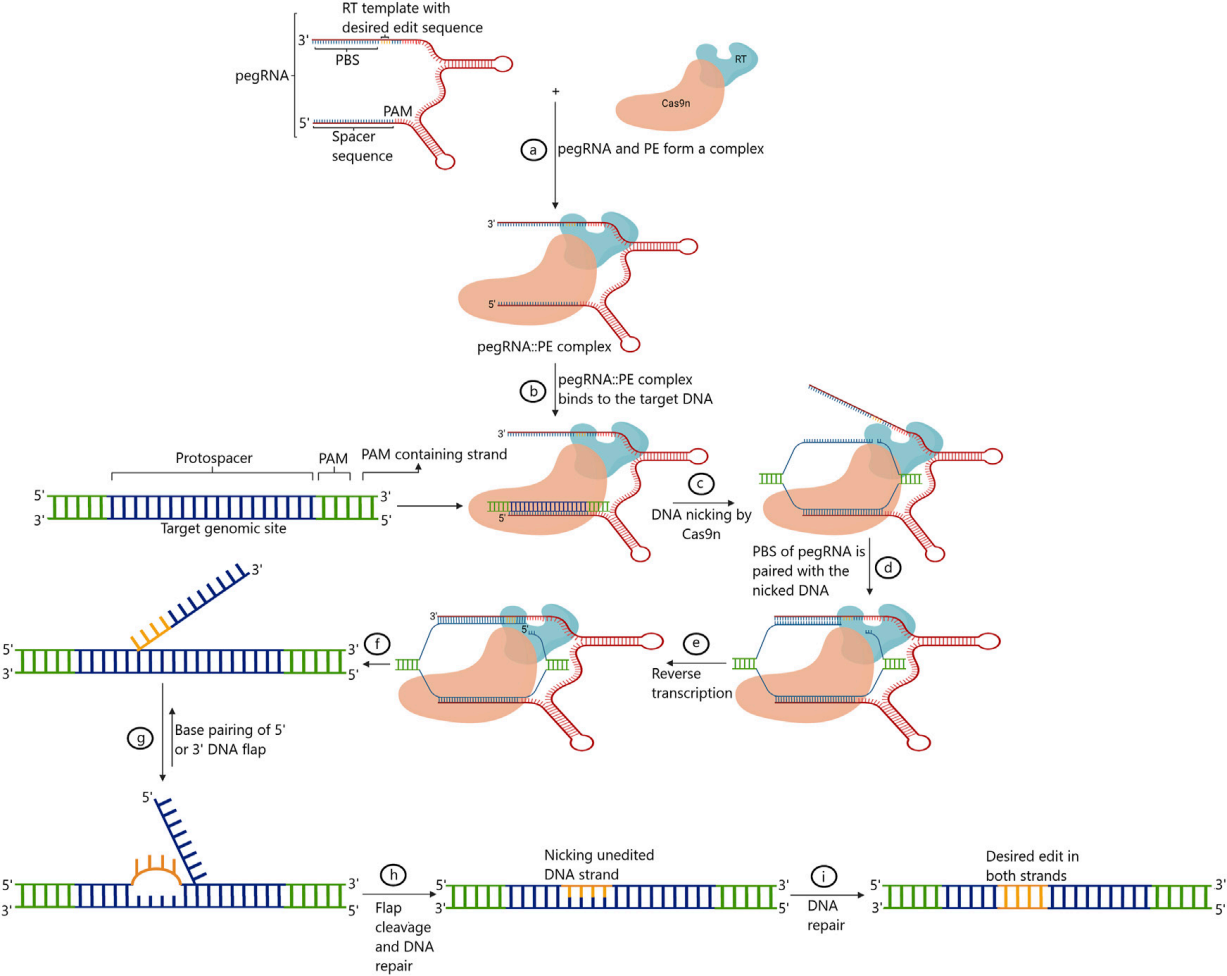
Diagrammatic representation of the Prime editing. sgRNA: single-guide RNA; Cas9n: Cas9 nickase; PAM: protospacer adjacent motif; PBS: primer binding site; RT: reverse transcriptase; pegRNA: prime editing guide RNA; PE: prime editor (adapted from Hassan et al. 2020).

Research has successfully validated three generations of primer editors (PEs), PE1, PE2, and PE3, in humans so far. In PE1, the first-generation PEs, wild-type reverse transcriptase from commercial Moloney murine leukemia virus (M-MLV) fused to the C terminus of the Cas9 (H840A) nickase was used, triggered by the expression of pegRNA in a distinct plasmid. As mentioned earlier, pegRNA harbors a spacer sequence to recognize and bind to the intended target site. In addition, pegRNA carries an 8–15 nt of PBS and a template sequence to drive RT. However, the template sequence also contains a customized, altered DNA sequence to be incorporated at the intended site. The efficiency of this PE is largely determined by PBS length. Generally, 8–16 nt PBS length has been found to deliver results with increased efficiency (Anzalone et al., 2019). In an attempt to further increase the efficiency of this PE, numerous variants of M-MLV RT have been used. These variants were generated by inducing mutations in M-MLV RT. These mutations were found to alter processivity, thermostability, RNaseH activity, and DNA–RNA substrate affinity. In developing second-generation prime editors, PE2 an RT with five mutations (D200N, L603W, T330P, T306K, and W313F), when fused with the nickase, was found to increase the efficiency of the GE by 1.6–5.1 fold (Sretenovic and Qi 2022). The use of PE2 was found to hinder the efficiency primarily due to two factors. Firstly, the choice of single-stranded overhangs called “flaps” between unedited and edited to be paired with the native unmodified DNA strand. Secondly, choosing DNA strands as a template for DNA repair between unedited and edited was rather random (Gaudelli et al., 2017; Sretenovic and Qi, 2022). Many studies have shown that the introduction of nick in the unmodified strand enhanced the editing efficiency in both plants and animal cells (Komor et al., 2016; Gaudelli et al., 2017; Zong et al., 2017). Hence, to generate third-generation prime editors, PE3, nickase employed was used with an additional sgRNA to simultaneously nick the other complementary strand (Anzalone et al., 2019). This strategy enhanced the editing efficiency to introduce point mutations three-fold (Anzalone et al., 2019). With the use of the same protospacer, off-target instances were found much lower for PEs in comparison to the use of Cas9 (Jiang et al., 2021; Jiang et al., 2022). The increased efficiency of the prime editor is attributed to multiple DNA hybridization events that occur with the use of PEs. At first, the intended genomic DNA and spacer of the pegRNA hybridize. Next, hybridization occurs between the target sequence in the genomic DNA and the PBS of the pegRNA, adding to the sequence specificity of the system. Finally, the target DNA also hybridizes with the edited DNA, which further adds another layer of sequence specificity to the system (Jiang et al., 2021; Jiang et al., 2022). On the contrary, in a regular CRISPR/Cas9 system, only one step of hybridization occurs between the sgRNA and the target genomic DNA occurs (Jiang et al., 2022; Zhuang et al., 2022). Figure 4 presents a schematic representation of the working mechanism of the prime editing methodology that has been employed for GE.

The success of prime editing protocols hinges on optimizing critical parameters such as transformation system, selection of suitable vectors, design of prime editor cassettes (nuclease/nickase), structure/sequence of, for example, pegRNA, sgRNA, codon optimization of the vector constructs, promoters, use of novel/engineered endonuclease, ribozymes, reverse transcriptase, targeted genes, and method/s of detection. *Agrobacterium*-mediated transformation and floral dip agroinfiltration are the preferred modes of gene transfer as single copy inserts are efficiently achieved. However, other methods such as electroporation, PEG-mediated gene uptake, microinjection, and particle bombardment have been tested in different plants and are now expanding rapidly to include monocots (rice and maize), dicots (*Arabidopsis*, *Nicotiana benthamiana*, potato, and tomato), and even the bryophyte, *Physcomitrium patens* (Perroud et al., 2022) that is well known for incorporating DNA into specific genomic sites due to its innately high frequencies of homologous recombination (Rensing et al., 2020).

Researchers have been experimenting extensively with the precise modeling of the molecular tool kit for high efficiency and specificity in several plants. As mentioned earlier, three versions of prime editors (PE1, PE2, and PE3) have been tested since 2019 in human and plant cells. The versions vary in the use of nickase, type of reverse transcriptase, position (C terminal or N-terminal fusion with nickase), length of the prime binding site, and types of editing predicted (Jiang et al., 2022). Promoters driving the expression of the prime editor apoprotein and the gRNAs play an important role in the overall scheme of prime editing in taxa and target gene of choice (Sretenovic and Qi 2022). Target sites have been categorized as type I and type II based on the position of the edit concerning the nicking site. If the edit is within 1–6 bp downstream of the pegRNA nicking site, then higher editing efficiencies are observed compared to the type II targets, where the targeted edit position(s) are 7–17 bp downstream of the pegRNA nicking site (Sretenovic and Qi, 2022). The editing efficiencies of the same vectors thus vary with the target genes. This was reported in rice, where the prime editor Sp-PE3 and gRNA were successful in introducing an S627N mutation in the endogenous *ALS* (acetolactate synthase) but were unsuccessful in editing the *APO1* (aberrant panicle organization) gene (Hua et al., 2020a). It was also successfully induced and present in regenerants. Three endogenous genes (*GAI*, *ALS2*, and *PDS1*) from tomato were tested for prime editing by PE3 strategy using an optimized prime editor. Prime editing frequencies of 0.025%–1.66% were observed in four pegRNAs out of seven tested, comparable to rice editing frequencies (Lu et al., 2020). Three genes (*OsPDS*, *OsACC1*, and *OsWx*) were used as targets to test the pPE2 system. Using the t-RNA processing strategy was also used to target a rice endogenous 5-enolpyruvylshikimate-3-phosphate synthase (EPSPS) gene (*OsEPSPS*) for prime editing to confer glyphosate resistance. A peg RNA with gRNA (59 bp RT, 13 nt PBS) and a second gRNA with the ability to nick at position 66 downstream were synthesized that could introduce triple mutations. For this gene-editing, the prime editing efficiency was 2.22% with both homozygous and heterozygous lines in rice (Li et al., 2020c). The pPPEM construct was tested in rice protoplasts, targeting gene *OsSULTR3*, six at two different edits for the bacterial leaf streak disease susceptibility. The editing efficiencies ranged from 0.7 to 2.2%. Besides editing endogenous genes, editing the transgenic reporter gene—fluorescent protein gene *EGFP* by SpPE2, SpPE3, and SaPE3—was tested in rice calli. The inactive insert was edited to active form successfully by SpPE3 at higher efficiencies than SpPE2, and none were observed with SaPE3, even though Sa compatible Cas9 and pegRNAs are required for efficient editing.

The prime-editing gRNAs of diverse structures with varied PBS and RT lengths and nicking position of gRNAs have also been reported to affect the prime editing efficiency (Xu et al., 2020; Hua et al., 2020a; Tang et al., 2020; Butt et al., 2020). Optimization of the melting temperature (Tm) of the PBS to around 30°C coupled with a dual-pegRNA strategy in plants (Lin et al., 2020) drastically increased the editing efficiencies by 17-fold in rice protoplasts, although stable expression and transmission of the edits remain to be seen. Inclusion of the t-RNA processing system (Xie et al., 2015) allows for the generation of multiple gRNAs that allow for “multiplex GE.”

Detection of editing relies on the rates of transformation coupled with the rate of editing. Several studies have reported the co-transfection of T-DNA-containing vectors with the transgene and the PE vectors harboring the editor and the edit. The targeted sites are usually PCR amplified from the genomic DNA isolated from transformed plants and sequenced to identify the edits. Most researchers have done Sanger’s sequencing, although the HRM-High Resolution Melting analysis has been included before sequencing by Perroud et al. (2022). Hi-TOM (high-throughput tracking of mutations) was used by Xu et al. (2022) in maize and rice.

Different selection and counter-selection strategies have been tested for the selection of transformed/edited cells. Perroud et al. (2022) have tested the use of APT/APRT (adenine phosphoribosyl transferase) enzyme that catalyzes the conversion of adenine to AMP in *Physcomitrium*. This enzyme can convert 2-fluoroadenine (2FA) supplemented in the culture medium into a toxic 2-fluoro AMP counter selective compound. Thus, if the editing vectors are successful, the APRT is mutated and the cells can grow and regenerate into plants on the 2FA medium. The DNA from these plants is further analyzed to detect edited sequences. In potato, the widely used acetolactate synthase (ALS) has been used for selection. ALS confers resistance to several herbicides, particularly chlorsulfuron, and the specific amino acid change in StALS Pro-187/186 to serine was targeted. In addition, the primary selection of transgenics was on kanamycin. A PE-PE2 system was designed by fusing hygromycin phosphotransferase (Hpt) to the C-terminus of the nSpCas9-M-MLV region with P2A, a self-cleaving 2A peptide, driven by Ubiquitin promoter of maize. PE-PE2 increased the editing efficiency by about threefold for three pegRNAs and gave improved editing frequencies (Perroud et al., 2022).

The ability to introduce both transversions and transitions is by far the most significant attribute of prime editing technology. In addition, PEs have been found to successfully introduce insertions, deletions, transitions, and transversions (Anzalone et al., 2019). Perroud et al. (2022) reported that 0.06% of transformed protoplasts of *Physcomitrium* were edited, which is less than the standard Cas9 mediated and base editing mutagenic strategies. However, the edit’s specificity is higher than CRISPR/Cas systems, and off-targets are few or none. Substitutions, insertions, and deletions have been observed in the different taxa using the varied versions of prime editors.

The editing efficiency was similar in PE2- and PE3-based vectors in *Physcomitrium*, whereas in potato, same PE3 constructs failed to edit the *ALS* gene, which could be edited by PE2-based vectors albeit at low frequencies. In rice, editing efficiencies were between 1.55% and 31.3% (Hua et al., 2020b; Butt et al., 2020; Li et al., 2020d; Lin et al., 2020; Tang et al., 2020; Xu et al., 2020). The editing efficiencies ranged from 0.7% to 2.2%. Overall, the PE3 strategies were less efficient in plant cells than animal cells. However, further modifications and adaptation of the technique would standardize prime editing for more crop systems. Wang et al. (2021) have reported insertion of up to 66 bases in *Arabidopsis* protoplasts, which is a four-fold increase over the 15-base insertion reported in rice. For prime editing in dicots and monocots, easy-use vectors on PE2 and PE3 strategies have been created, named pPPED and pPPEM (Wang et al., 2021). They have designed a pPEG cassette for insertion of peg RNA or sgRNA, and then pPEG is inserted in the vectors PPEM or PPED. The pPPED vector was targeted in *Arabidopsis*. Editing efficiency is thus influenced by the length of reverse transcriptase and primer-binding site in the designed pegRNAs and sgRNAs.

In addition to the biological parameters (plant taxa, molecular toolkit, transformation, and regeneration system), the physical temperature parameter has a profound impact on the editing frequencies. Because the efficiency of the M-MLV reverse transcriptase is enhanced at higher temperatures, 32°C and 37°C were tested, but no significant differences were reported. However, the temperature variations were also tried in prime editing (PPE) systems at 26°C and 37°C in rice, giving significantly higher editing activity at 37°C (Lin et al., 2020).

In summary, the modifications in the design of constructs, particularly to avoid by-products resulting from the scaffold of the pegRNAs and reduction of off-targets, have been found to increase the editing efficiencies. Gao (2015) suggested the shift from a knock-out strategy to a knock-in strategy by employing the homologous recombination process of DNA repair to increase targeted mutagenesis. This has been incorporated as a key attribute in the prime editing technology. Among the diverse strategies designed to achieve targeted mutagenesis, prime editing is a landmark advancement in methods achieving increased efficiency and reduced off-target effects. This method, for the first time, presented an efficient strategy to introduce all the 12-point mutations. With the availability of many diverse vectors (editors and pegRNAs) developed by the different research groups and web-based design algorithms available (Peg-finder, PE-Designer /PE-Analyzer, pegIT, PrimeDesign, and PlantPegDesigner), the deployment of this technique is at the threshold of revolutionizing precision breeding of crop plants. As most of the genes of importance rely on altering a few and specific nucleotide changes to confer traits rather than large-scale alteration of genes, prime editing presents an opportunity to drive the development of gene editing platforms that are precise, effective, and elegant.

## 8 Conclusion

Under the scenario of ever-rising food demands and climate change, there is tremendous pressure on scientists and breeders to speed up the development of climate-resilient-high-yielding cultivars. The application of molecular breeding approaches has achieved great success in accelerating performance gains in various crops in the past decade. However, the need of the hour is to integrate new biotechnological methods and technologies in the existing breeding programs to further realize genetic gains. The unprecedented advances made in GE technologies have shown great potential in genetic enhancement and boosting crop production. This review highlights how newly evolved CRISPR/Cas systems have successfully brought about a paradigm shift in crop improvement programs. There has been a significant advancement in understanding the functions of gene complexes underpinning complex traits, which was extremely daunting using the existing gene discovery approaches. The efficient use of GE tools in manipulating complex traits, especially in polyploid crops, has now become feasible, especially when used in combination with the next-generation sequencing platforms.

Despite the substantial deployment of the CRISPR/Cas platform in developing crops with desired traits, studies demonstrating the translation of the laboratory-based results into the field have been anecdotal. In addition to being relevant at the genome level, the improved traits must also be realized in the field without any trade-offs or counter effects on other traits of importance. Additionally, any genome strategy developed should pose no threat to the environment and should be able to reduce the application of pesticides and fertilizers. One of the major challenges in developing cultivars by the GE route is rooted in low transformation and regeneration efficiencies. Numerous agronomically important crops such as sunflower, cotton, and many others either have long transformation protocols with low efficiencies or are outrightly recalcitrant. In addition, in crops where transformation protocols have been established, regeneration efficiencies remain low, making the application of GE strategies challenging.

Furthermore, public acceptance of GE-modified crops has not come of age yet. A common misconception about these crops adversely affecting health and the environment has led many farmers to avoid reaping benefits from growing these crop cultivars. This bias automatically trickles down to the consumers and, in turn, results in limited acceptance of these crops for public consumption. Therefore, we believe, scientists across the globe need to ensure a healthy flow of information using present-day outreach tools, including social media, to educate the consumers about the differences between transgenic approaches and the risks and benefits of using modern GE-modified crops.

Although GE platforms are radically different, precise, and superior to traditional transgenic approaches, at the moment, these methods still go through governmental scrutiny and assessment in many countries. Nonetheless, in the foreseeable future, new-age GE platforms in plants are contemplated to be employed as a tool for efficiently engineering the majority of crop plants. We expect and hope that these methods can be integrated into breeding programs globally with relatively lesser regulatory procedures compared to conventional transgenic approaches. The development of these measures will need comparable attention and consistent research efforts to continually assess developed crop varieties on various climatic and genomic parameters, especially in our present-day rapidly changing climate and pest pressure.

## 9 Future directions

The evolution of various GE platforms has made it possible for molecular biologists to precisely target gene(s) of interest. Primarily, only CRISPR/Cas has been used for gene editing. Only recently, techniques such as epigenome editing, prime editing, and base editing have been used for gene editing. These techniques are powerful alternative strategies that have been developed for gene editing in plants. However, glaring challenges still exist that continue to impede the goals of achieving sustainable crop production. These challenges stem from the complexity of both endogenous and exogenous cues in plant development, making it nearly impossible for any single GE platform to deliver efficiently. Present-day advances in GE protocols need to be primed toward generating platforms that are more precise, efficient, accurate, and, most importantly, feasible. At first, no off-target silencing should result from using these methods. Secondly, the delivery and results obtained in crop plants should not vary from species to species. In addition, the genomic changes should be traceable in future generations with precision and also remain feasible with respect to cost and labor. Lastly, at present, we need more dynamic regulatory measures in place to ease the development and use of these platforms in crop improvement programs.

## References

[B1] Akama K. Akter N. Endo H. Kanesaki M. Endo M. Toki S. (2020). An *in vivo* targeted deletion of the calmodulin-binding domain from rice glutamate decarboxylase 3 (Os GAD3) increases γ-aminobutyric acid content in grains. Rice 13 (1), 20. 10.1186/s12284-020-00380-w 32180062 PMC7076103

[B2] Ali Z. Ali S. Tashkandi M. Zaidi S. S. Mahfouz M. M. (2016). CRISPR/Cas9- mediated immunity to geminiviruses: Differential interference and evasion. Sci. Rep. 6, 26912. 10.1038/srep26912 27225592 PMC4881029

[B192] Anders C. Niewoehner O. Duerst A. Jinek M. (2014). Structural basis of PAM-dependent target DNA recognition by the Cas9 endonuclease. Nature 513 (7519), 569–573. 25079318 10.1038/nature13579 PMC4176945

[B3] Amitai G. Sorek R. (2016). CRISPR–Cas adaptation: insights into the mechanism of action. Nat. Rev. Microbiol. 14 (2), 67–76. 10.1038/nrmicro.2015.14 26751509

[B4] Anzalone A. V. Randolph P. B. Davis J. R. Sousa A. A. Koblan L. W. Levy J. M. (2019). Search-and-replace genome editing without double-strand breaks or donor DNA. Nature 576, 149–157. 10.1038/s41586-019-1711-4 31634902 PMC6907074

[B5] Barrangou R. Fremaux C. Deveau H. Richards M. Boyaval P. Moineau S. (2007). CRISPR provides acquired resistance against viruses in prokaryotes. Science 315, 1709–1712. 10.1126/science.1138140 17379808

[B6] Ben Shlush I. Samach A. Melamed-Bessudo C. Ben-Tov D. Dahan-Meir T. Filler- Hayut S. (2021). CRISPR/Cas9 induced somatic recombination at the CRTISO locus in tomato. Genes 12 (1), 59. 10.3390/genes12010059 PMC7824628 33396568

[B193] Bharat S. S. Li S. Li J. Yan L. Xia L. (2020). Base editing in plants: Current status and challenges. Crop J. 8 (3), 384–395.

[B7] Bhowmik P. Ellison E. Polley B. Bollina V. Kulkarni M. Ghanbarnia K. (2018). Targeted mutagenesis in wheat microspores using CRISPR/Cas9. Sci. Rep. 8 (1), 6502. 10.1038/s41598-018-24690-8 29695804 PMC5916876

[B8] Boch J. Bonas U. (2010). Xanthomonas AvrBs3 family-type III effectors: Discovery and function. Annu. Rev. Phytopathol. 418, 419–436. 10.1146/annurev-phyto-080508-081936 19400638

[B9] Bollen Y. Post J. Koo B. K. Snippert H. J. (2018). How to create state-of-the-art genetic model systems: strategies for optimal CRISPR-mediated genome editing. Nucleic Acids Res. 46 (13), 6435–6454. 10.1093/nar/gky571 29955892 PMC6061873

[B10] Brocken D. J. W. Tark-Dame M. Dame R. T. (2018). dCas9: A versatile tool for epigenome editing. Curr. Issues Mol. Biol. 26, 15–32. 10.21775/cimb.026.015 28879853

[B11] Butt H. Rao G. S. Sedeek K. Aman R. Kamel R. Mahfouz M. (2020). Engineering herbicide resistance via prime editing in rice. Plant Biotechnol. J. 18, 2370–2372. 10.1111/pbi.13399 32415890 PMC7680537

[B12] Calvache C. Vazquez-Vilar M. Selma S. Uranga M. Fernández-del-Carmen A. Daròs J. A. (2022). Strong and tunable anti-CRISPR/Cas activities in plants. Plant Biotechnol. J. 20 (2), 399–408. 10.1111/pbi.13723 34632687 PMC8753356

[B13] Čermák T. Baltes N. J. Čegan R. Zhang Y. Voytas D. F. (2015). High-frequency, precise modification of the tomato genome. Genome Biol. 16 (1), 232. 10.1186/s13059-015-0796-9 26541286 PMC4635538

[B14] Chandrasekaran J. Brumin M. Wolf D. Leibman D. Klap C. Pearlsman M. (2016). Development of broad virus resistance in non-transgenic cucumber using CRISPR/Cas9 technology. Mol. Plant Pathol. 17, 1140–1153. 10.1111/mpp.12375 26808139 PMC6638350

[B15] Chaudhuri A. Halder K. Datta A. (2022). Classification of CRISPR/Cas system and its application in tomato breeding. Theor. Appl. Genet. 135, 367. 10.1007/s00122-021-03984-y 34973111 PMC8866350

[B194] Choi M. Yun J. Y. Kim J. H. Kim J. S. Kim S. T. (2021). The efficacy of CRISPR-mediated cytosine base editing with the RPS5a promoter in Arabidopsis thaliana. Sci. Rep. 11, 8087. 10.1038/s41598-021-87669-y 33850267 PMC8044221

[B16] Chen L. Park J. E. Paa P. Rajakumar P. D. Prekop H. T. Chew Y. T. (2021). Programmable C: G to G: C genome editing with CRISPR-cas9- directed base excision repair proteins. Nat. Commun. 12 (1), 1384. 10.1038/s41467-021-21559-9 33654077 PMC7925527

[B17] Chen Y. Wang Z. Ni H. Xu Y. Chen Q. Jiang L. (2017). CRISPR/Cas9-mediated base-editing system efficiently generates gain-of-function mutations in Arabidopsis. Sci. China. Life Sci. 60 (5), 520–523. 10.1007/s11427-017-9021-5 28303459

[B18] Cheng H. Hao M. Ding B. Mei D. Wang W. Wang H. (2021). Base editing with high efficiency in allotetraploid oilseed rape by A3A-PBE system. Plant Biotechnol. J. 19 (1), 87–97. 10.1111/pbi.13444 32640102 PMC7769242

[B19] Cheng T. L. Li S. Yuan B. Wang X. Zhou W. Qiu Z. (2019). Expanding C-T base editing toolkit with diversified cytidine deaminases. Nat. Commun. 10, 3612. 10.1038/s41467-019-11562-6 31399578 PMC6689024

[B20] Cong L. Ran F. A. Cox D. Lin S. Barretto R. Habib N. (2013). Multiplex genome engineering using CRISPR/Cas systems. Science 339 (6121), 819–823. 10.1126/science.1231143 23287718 PMC3795411

[B21] Cong L. Zhou R. Kuo Y. C. Cunniff M. Zhang F. (2012). Comprehensive interrogation of natural TALE DNA-binding modules and transcriptional repressor domains. Nat. Commun. 3, 968. 10.1038/ncomms1962 22828628 PMC3556390

[B22] D’Ambrosio C. Stigliani A. L. Giorio G. (2018). CRISPR/Cas9 editing of carotenoid genes in tomato. Transgenic Res. 27 (4), 367–378. 10.1007/s11248-018-0079-9 29797189

[B23] Datsenko K. A. Pougach K. Tikhonov A. Wanner B. L. Severinov K. Semenova E. (2012). Molecular memory of prior infections activates the CRISPR/Cas adaptive bacterial immunity system. Nat. Commun. 3, 945. 10.1038/ncomms1937 22781758

[B24] Deb S. Choudhury A. Kharbyngar B. Satyawada R. R. (2022). Applications of CRISPR/Cas9 technology for modification of the plant genome. Genetica, 150. 1–12. 10.1007/s10709-021-00146-2 35018532

[B25] Dong L. Qi X. Zhu J. Liu C. Zhang X. Cheng B. (2019). Supersweet and waxy: meeting the diverse demands for specialty maize by genome editing. Plant Biotechnol. J. 17 (10), 1853–1855. 10.1111/pbi.13144 31050154 PMC6737015

[B26] Dong O. X. Yu S. Jain R. Zhang N. Duong P. Q. Butler C. (2020). Marker-free carotenoid-enriched rice generated through targeted gene insertion using CRISPR-Cas9. Nat. Commun. 11 (1), 1178. 10.1038/s41467-020-14981-y 32132530 PMC7055238

[B195] Endo A. Masafumi M. Kaya H. Toki S. (2016). Efficient targeted mutagenesis of rice and tobacco genomes using Cpf1 from *Francisella novicida* . Sci. Rep. 6, 38169. 10.1038/srep38169 27905529 PMC5131344

[B196] Endo M. Mikami M. Endo A. Kaya H. Itoh T. Nishimasu H. (2019). Genome editing in plants by engineered CRISPR–Cas9 recognizing NG PAM. Nature Plants 5 (1), 14–17. 30531939 10.1038/s41477-018-0321-8

[B27] Fan S. Zhang L. Tang M. Cai Y. Liu J. Liu H. (2021). CRISPR/Cas9-targeted mutagenesis of the BnaA03. BP gene confers semi-dwarf and compact architecture to rapeseed (Brassica napus L.). Plant Biotechnol. J. 19 (12), 2383–2385. 10.1111/pbi.13703 34498373 PMC8633515

[B28] Fonfara I. Richter H. Bratovič M. Le Rhun A. Charpentier E. (2016). The CRISPR- associated DNA-cleaving enzyme Cpf1 also processes precursor CRISPR RNA. Nature 532, 517–521. 10.1038/nature17945 27096362

[B29] Gahlaut S. K. Savargaonkar D. Sharan C. Yadav S. Mishra P. Singh J. P. (2020). SERS platform for dengue diagnosis from clinical samples employing a hand held Raman spectrometer. Anal. Chem. 92 (3), 2527–2534. 10.1021/acs.analchem.9b04129 31909593

[B30] Gahlaut V. Zinta G. Jaiswal V. Kumar S. (2020). Quantitative epigenetics: A new avenue for crop improvement. Epigenomes 4, 25. 10.3390/epigenomes4040025 34968304 PMC8594725

[B31] Gallego-Bartolomé J. (2020). DNA methylation in plants: Mechanisms and tools for targeted manipulation. New Phytol. 227, 38–44. 10.1111/nph.16529 32159848

[B32] Gallego-Bartolomé J. Gardiner J. Liu W. Papikian A. Ghoshal B. Kuo H. Y. (2018). Targeted DNA demethylation of the *Arabidopsis* genome using the human TET1 catalytic domain. Proc. Natl. Acad. Sci. U. S. A. 115, E2125–E2134. 10.1073/pnas.1716945115 29444862 PMC5834696

[B33] Gallego-Bartolome J. Liu W. Kuo P. H. Feng S. Ghoshal B. Gardiner J. (2019). Co-targeting RNA polymerases IV and V promotes efficient de novo DNA methylation in *Arabidopsis* . Cell 176, 1068–1082. 10.1016/j.cell.2019.01.029 30739798 PMC6386582

[B197] Gao C. (2015). Genome editing in crops: from bench to field. Nat. Sci. Rev. 2 (1), 13–15.

[B34] Gao H. Gadlage M. J. Lafitte H. R. Lenderts B. Yang M. Schroder M. (2020). Superior field performance of waxy corn engineered using CRISPR–Cas9. Nat. Biotechnol. 38 (5), 579–581. 10.1038/s41587-020-0444-0 32152597

[B35] Gaudelli N. M. Komor A. C. Rees H. A. Packer M. S. Badran A. H. Bryson D. I. (2017). Programmable base editing of A·T to G·C in genomic DNA without DNA cleavage. Nature 551 (7681), 464–471. 10.1038/nature24644 29160308 PMC5726555

[B36] Gehrke J. M. Cervantes O. Clement M. K. Wu Y. Zeng J. Bauer D. E. (2018). An APOBEC3A-Cas9 base editor with minimized bystander and off-target activities. Nat. Biotechnol. 36 (10), 977–982. 10.1038/nbt.4199 30059493 PMC6181770

[B37] Ghoshal B. Picard C. L. Vong B. Feng S. Jacobsen S. E. (2021). CRISPR-based targeting of DNA methylation in *Arabidopsis thaliana* by a bacterial CG-specific DNA methyltransferase. Proc. Natl. Acad. Sci. U. S. A. 118, e2125016118. 10.1073/pnas.2125016118 34074795 PMC8201958

[B38] Giudice G. Moffa L. Varotto S. Cardone M. F. Bergamini C. De Lorenzis G. (2021). Novel and emerging biotechnological crop protection approaches. Plant Biotechnol. J. 19, 1495–1510. 10.1111/pbi.13605 33945200 PMC8384607

[B39] Grunewald J. Zhou R. H. Lareau C. A. Garcia S. P. Iyer S. Miller B. R. (2020). A dual-deaminase CRISPR base editor enables concurrent adenine and cytosine editing. Nat. Biotechnol. 38 (7), 861–864. 10.1038/s41587-020-0535-y 32483364 PMC7723518

[B40] Guilinger J. P. Thompson D. B. Liu D. R. (2014). Fusion of catalytically inactive Cas9 to FokI nuclease improves the specificity of genome modification. Nat. Biotechnol. 32 (6), 577–582. 10.1038/nbt.2909 24770324 PMC4263420

[B41] Guo H. Wu T. Li S. He Q. Yang Z. Zhang W. (2019). The methylation patterns and transcriptional responses to chilling stress at the seedling stage in rice. Int. J. Mol. Sci. 20, 5089. 10.3390/ijms20205089 PMC6829347 31615063

[B198] Han H. Wu Z. Zheng L. Han J. Zhang Y. Li J. (2022). Generation of a high-efficiency adenine base editor with TadA8e for developing wheat dinitroaniline-resistant germplasm. Crop J. 10 (2), 368–374.

[B199] Hassan M. Yuan G. Chen J. G. Tuskan G. A. Yang X. (2020). Prime editing technology and its prospects for future applications in plant biology research. BioDesign Res. 2020. 10.34133/2020/9350905 PMC10530660 37849904

[B42] Hess G. T. Fresard L. Han K. Lee C. H. Li A. Cimprich K. A. (2016). Directed evolution using dCas9-targeted somatic hypermutation in mammalian cells. Nat. Methods 13, 1036–1042. 10.1038/nmeth.4038 27798611 PMC5557288

[B43] Hong Y. Meng J. He X. Zhang Y. Liu Y. Zhang C. (2021). Editing miR482b and miR482c simultaneously by CRISPR/Cas9 enhanced tomato resistance to Phytophthora infestans. Phytopathology 111 (6), 1008–1016. 10.1094/PHYTO-08-20-0360-R 33258411

[B44] Hu L. Amoo O. Liu Q. Cai S. Zhu M. Shen X. (2020). Precision genome engineering through cytidine base editing in rapeseed (Brassica napus. L). Front. Genome Ed. 15, 605768. 10.3389/fgeed.2020.605768 PMC8525351 34713230

[B45] Hu X. Meng X. Liu Q. Li J. Wang K. (2018). Increasing the efficiency of CRISPR- Cas9-VQR precise genome editing in rice. Plant Biotechnol. J. 16 (1), 292–297. 10.1111/pbi.12771 28605576 PMC5785341

[B46] Hua K. Jiang Y. Tao X. Zhu J. K. (2020a). Precision genome engineering in rice using prime editing system. Plant Biotechnol. J. 18, 2167–2169. 10.1111/pbi.13395 32372479 PMC7589318

[B47] Hua K. Tao X. Liang W. Zhang Z. Gou R. Zhu J. K. (2020b). Simplified adenine base editors improve adenine base editing efficiency in rice. Plant Biotechnol. J. 18 (3), 770–778. 10.1111/pbi.13244 31469505 PMC7004905

[B48] Hua K. Tao X. Yuan F. Wang D. Zhu J. K. (2018). Precise A·T to G·C base editing in the rice genome. Mol. Plant 11 (4), 627–630. 10.1016/j.molp.2018.02.007 29476916

[B49] Hua K. Tao X. Zhu J. K. (2019). Expanding the base editing scope in rice by using Cas9 variants. Plant Biotechnol. J. 17 (2), 499–504. 10.1111/pbi.12993 30051586 PMC6335069

[B200] Hunziker J. Nishida K. Kondo A. Kishimoto S. Ariizumi T. Ezura H. (2020). Multiple gene substitution by Target-AID base-editing technology in tomato. Sci. Rep. 10, 20471. 10.1038/s41598-020-77379-2 33235312 PMC7686336

[B50] Huang H. Cui T. Zhang L. Yang Q. Yang Y. Xie K. (2020). Modifications of fatty acid profile through targeted mutation at BnaFAD2 gene with CRISPR/Cas9-mediated gene editing in Brassica napus. Theor. Appl. Genet. 133 (8), 2401–2411. 10.1007/s00122-020-03607-y 32448919

[B201] Ishino Y. Shinagawa H. Makino K. Amemura M. Nakata A. (1987). Nucleotide sequence of the iap gene, responsible for alkaline phosphatase isozyme conversion in Escherichia coli, and identification of the gene product. J. Bacteriol. 169 (12), 5429-5433. 10.1128/jb.169.12.5429-5433 3316184 PMC213968

[B51] Jaganathan D. Ramasamy K. Sellamuthu G. Jayabalan S. Venkataraman G. (2018). CRISPR for crop improvement: An update review. Front. Plant Sci. 9, 985. 10.3389/fpls.2018.00985 30065734 PMC6056666

[B52] Jiang F. Doudna J. A. (2017). CRISPR–Cas9 structures and mechanisms. Annu. Rev. Biophys. 46, 505–529. 10.1146/annurev-biophys-062215-010822 28375731

[B53] Jiang T. Zhang X. O. Weng Z. Xue W. (2022). Deletion and replacement of long genomic sequences using prime editing. Nat. Biotechnol. 40 (2), 227–234. 10.1038/s41587-021-01026-y 34650270 PMC8847310

[B54] Jiang T. Zhang X. O. Weng Z. Xue W. (2021). Programming large target genomic deletion and concurrent insertion via a prime editing-based method: Pedar. USA: Cold Spring Harbor Laboratory. *bioRxiv* .

[B55] Jinek M. Chylinski K. Fonfara I. Hauer M. Doudna J. A. Charpentier E. (2012). A programmable dual- RNA– guided DNA endonuclease in adaptive bacterial immunity. Science 337 (6096), 816–821. 10.1126/science.1225829 22745249 PMC6286148

[B56] Johnson L. M. Du J. Hale C. J. Bischof S. Feng S. Chodavarapu R. K. (2014). SRA- and SET-domain-containing proteins link RNA polymerase V occupancy to DNA methylation. Nature 507, 124–128. 10.1038/nature12931 24463519 PMC3963826

[B57] Kakoulidou I. Avramidou E. V. Baránek M. Brunel-Muguet S. Farrona S. Johannes F. (2021). Epigenetics for crop improvement in times of global change. Biology 10, 766. 10.3390/biology10080766 34439998 PMC8389687

[B58] Kantor A. McClements M. E. MacLaren R. E. (2020). CRISPR-Cas9 DNA base- editing and prime-editing. Int. J. Mol. Sci. 21 (17), 6240. 10.3390/ijms21176240 PMC7503568 32872311

[B202] Karginov F. V. Hannon G. J. (2010). The CRISPR system: Small RNA-guided defense in bacteria and archaea. Mol. Cell 37 (1), 7–19. 10.1016/j.molcel.2009.12.033 20129051 PMC2819186

[B59] Kashojiya S. Lu Y. Takayama M. Komatsu H. Minh L. H. T. Nishida K. (2022). Modification of tomato breeding traits and plant hormone signaling by target-AID, the genome-editing system inducing efficient nucleotide substitution. Hortic. Res. 9, uhab004. 10.1093/hr/uhab004 PMC8795821 35043178

[B60] Kaya H. Ishibashi K. Toki S. (2017). A split *Staphylococcus aureus* Cas9 as a compact genome-editing tool in plants. Plant Cell Physiol. 58 (4), 643–649. 10.1093/pcp/pcx034 28371831 PMC5444561

[B61] Khan I. Khan S. Zhang Y. Zhou J. Akhoundian M. Jan S. A. (2021). CRISPR-Cas technology-based genome editing for modification of salinity stress tolerance responses in rice (Oryza sativa L.). Mol. Biol. Rep. 48 (4), 3605–3615. 10.1007/s11033-021-06375-0 33950408

[B62] Kim H. Kim S. T. Ryu J. Kang B. C. Kim J. S. Kim S. G. (2017). CRISPR/Cpf1- mediated DNA-free plant genome editing. Nat. Commun. 8, 14406. 10.1038/ncomms14406 28205546 PMC5316869

[B63] Kim Y. G. Cha J. Chandrasegaran S. (2007). Hybrid restriction enzymes: zinc finger fu- sions to fok I cleavage domain. Proc. Natl. Acad. Sci. U. S. A. 93, 1156–1160. 10.1073/pnas.93.3.1156 PMC40048 8577732

[B64] Kleinstiver B. P. Prew M. S. Tsai S. Q. Nguyen N. T. Topkar V. V. Zheng Z. (2015). Broadening the targeting range of *Staphylococcus aureus* CRISPR-Cas9 by modifying PAM recognition. Nat. Biotechnol. 33 (12), 1293–1298. 10.1038/nbt.3404 26524662 PMC4689141

[B65] Komivi D. Marie A. M. Rong Z. Qi Z. Mei Y. Ndiaga C. (2018). The contrasting response to drought and waterlogging is underpinned by divergent DNA methylation programs associated with transcript accumulation in sesame. Plant Sci. 277, 207–217. 10.1016/j.plantsci.2018.09.012 30466587

[B66] Komor A. C. Kim Y. B. Packer M. S. Zuris J. A. Liu D. R. (2016). Programmable editing of a target base in genomic DNA without double-stranded DNA cleavage. Nature 533 (7603), 420–424. 10.1038/nature17946 27096365 PMC4873371

[B67] Konermann S. Brigham M. D. Trevino A. E. Hsu P. D. Heidenreich M. Cong L. (2013). Optical control of mammalian endogenous transcription and epigenetic states. Nature 500 (7463), 472–476. 10.1038/nature12466 23877069 PMC3856241

[B68] Koonin E. V. Makarova K. S. (2019). Origins and evolution of CRISPR-Cas systems. Philos. Trans. R. Soc. Lond. B Biol. Sci. 374 (1772), 20180087. 10.1098/rstb.2018.0087 30905284 PMC6452270

[B203] Kuang Y. Li S. Ren B. Yan F. Spetz C. Li X. (2020). Base-editing-mediated artificial evolution of OsALS1 in planta to develop novel herbicide-tolerant rice germplasms. Mol. Plant 13 (4), 565–572. 32001363 10.1016/j.molp.2020.01.010

[B69] Kumar A. Singh P. Sharma S. Pandey A. K. (2021). “Shifting paradigm towards the crops: From model plants to crops and employing the genome engineering to target traits,” in Agricultural Biotechnology: Latest research and trends (Singapore: Springer), 511–535.

[B70] Kumar S. Beena A. S. Awana M. Singh A. (2017). Salt-induced tissue-specific cytosine methylation downregulates expression of HKT genes in contrasting wheat (*Triticum aestivum* L.) genotypes. DNA Cell Biol. 36, 283–294. 10.1089/dna.2016.3505 28384069 PMC5385449

[B71] Kurt I. C. Zhou R. Iyer S. Garcia S. P. Miller B. R. Langner L. M. (2021). CRISPR C-to-G base editors for inducing targeted DNA transversions in human cells. Nat. Biotechnol. 39, 41–46. 10.1038/s41587-020-0609-x 32690971 PMC7854778

[B72] Lee C. M. Cradick T. J. Bao G. (2016). The Neisseria meningitidis CRISPR-Cas9 system enables specific genome editing in mammalian cells. Mol. Ther. 24 (3), 645–654. 10.1038/mt.2016.8 26782639 PMC4786937

[B204] Li C. Zong Y. Wang Y. Jin S. Zhang D. Song Q. (2018). Expanded base editing in rice and wheat using a Cas9-adenosine deaminase fusion. Genome Biol. 19 (1), 1–9. 29807545 10.1186/s13059-018-1443-z PMC5972399

[B73] Li C. Zhang R. Meng X. Chen S. Zong Y. Lu C. (2020). Targeted, random mutagenesis of plant genes with dual cytosine and adenine base editors. Nat. Biotechnol. 38 (7), 875–882. 10.1038/s41587-019-0393-7 31932727

[B74] Li G. Sretenovic S. Eisenstein E. Coleman G. Qi Y. (2021). Highly efficient C-to-T and A-to-G base editing in a Populus hybrid. Plant Biotechnol. J. 19 (6), 1086–1088. 10.1111/pbi.13581 33742755 PMC8196628

[B75] Li H. Li J. Chen J. Yan L. Xia L. (2020a). Precise modifications of both exogenous and endogenous genes in rice by prime editing. Mol. Plant 13, 671–674. 10.1016/j.molp.2020.03.011 32222486

[B76] Li H. Li J. Chen J. Yan L. Xia L. (2020b). Precise modifications of both exogenous and endogenous genes in rice by prime editing. Mol. Plant 13, 671–674. 10.1016/j.molp.2020.03.011 32222486

[B77] Li H. Zhao X. Dai H. Wu W. Mao W. Zhang Z. (2012). Tissue culture responsive microRNAs in strawberry. Plant Mol. Biol. Rep. 30, 1047–1054. 10.1007/s11105-011-0406-2

[B79] Li J. Wang Z. He G. Ma L. Deng X. W. (2020c). CRISPR/Cas9-mediated disruption of TaNP1 genes results in complete male sterility in bread wheat. J. Genet. Genomics 47 (5), 263–272. 10.1016/j.jgg.2020.05.004 32694014

[B80] Li J. Yang D. L. Huang H. Zhang G. He L. Pang J. (2020d). Epigenetic memory marks determine epiallele stability at loci targeted by de novo DNA methylation. Nat. Plants 6, 661–674. 10.1038/s41477-020-0671-x 32514141

[B81] Li J. Zhang X. Sun Y. Zhang J. Du W. Guo X. (2018). Efficient allelic replacement in rice by gene editing: a case study of the *NRT1.1B* gene. J. Integr. Plant Biol. 60, 536–540. 10.1111/jipb.12650 29575650

[B82] Li M. Li X. Zhou Z. Wu P. Fang M. Pan X. (2016). Reassessment of the four yield-related genes Gn1a, DEP1, GS3, and IPA1 in rice using a CRISPR/Cas9 system. Front. Plant Sci. 7, 377. 10.3389/fpls.2016.00377 27066031 PMC4811884

[B83] Li P. Li Y. J. Zhang F. J. Zhang G. Z. Jiang X. Y. Yu H. M. (2017). The Arabidopsis UDP-glycosyltransferases UGT79B2 and UGT79B3, contribute to cold, salt and drought stress tolerance via modulating anthocyanin accumulation. Plant J. 89, 85–103. 10.1111/tpj.13324 27599367

[B84] Li R. Char S. N. Liu B. Liu H. Li X. Yang B. (2021). High-efficiency plastome base editing in rice with TAL cytosine deaminase. Mol. Plant 14 (9), 1412–1414. 10.1016/j.molp.2021.07.007 34265443

[B85] Li R. Li R. Li X. Fu D. Zhu B. Tian H. (2017). Multiplexed CRISPR/Cas9-mediated metabolic engineering of γ-aminobutyric acid levels in Solanum lycopersicum. Plant Biotechnol. J. 16, 415–427. 10.1111/pbi.12781 28640983 PMC5787826

[B86] Li R. Liu C. Zhao R. Wang L. Chen L. Yu W. (2019). CRISPR/Cas9-Mediated SlNPR1 mutagenesis reduces tomato plant drought tolerance. BMC Plant Biol. 19 (1), 38. 10.1186/s12870-018-1627-4 30669982 PMC6341727

[B87] Li R. Zhang L. Wang L. Chen L. Zhao R. Sheng J. (2018). Reduction of tomato-plant chilling tolerance by CRISPR–Cas9-mediated SlCBF1 mutagenesis. J. Agric. Food Chem. 66 (34), 9042–9051. 10.1021/acs.jafc.8b02177 30096237

[B90] Liang Y. Xie J. Zhang Q. Wang X. Gou S. Lin L. (2022). AGBE: a dual deaminase-mediated base editor by fusing CGBE with ABE for creating a saturated mutant population with multiple editing patterns. Nucleic Acids Res. 50 (9), 5384–5399. 10.1093/nar/gkac353 35544322 PMC9122597

[B92] Lin Q. Zong Y. Xue C. Wang S. Jin S. Zhu Z. (2020). Prime genome editing in rice and wheat. Nat. Biotechnol. 38, 582–585. 10.1038/s41587-020-0455-x 32393904

[B93] Liu H. Ding Y. Zhou Y. Jin W. Xie K. Chen L. L. (2017). CRISPR-P 2.0: an improved CRISPR-cas9 tool for genome editing in plants. Mol. Plant 10 (3), 530–532. Epub 2017 Jan 13. 10.1016/j.molp.2017.01.003 28089950

[B94] Liu L. Gallagher J. Arevalo E. D. Chen R. Skopelitis T. Wu Q. (2021). Enhancing grain-yield-related traits by CRISPR–Cas9 promoter editing of maize CLE genes. Nat. Plants 7 (3), 287–294. 10.1038/s41477-021-00858-5 33619356

[B95] Liu L. Zhang J. Xu J. Li Y. Guo L. Wang Z. (2020). CRISPR/Cas9 targeted mutagenesis of SlLBD40, a lateral organ boundaries domain transcription factor, enhances drought tolerance in tomato. Plant Sci. 301, 110683. 10.1016/j.plantsci.2020.110683 33218644

[B96] Liu X. Homma A. Sayadi J. Yang S. Ohashi J. Takumi T. (2016). Sequence features associated with the cleavage efficiency of CRISPR/Cas9 system. Sci. Rep. 6, 19675. 10.1038/srep19675 26813419 PMC4728555

[B98] Liu X. Zhang S. Jiang Y. Yan T. Fang C. Hou Q. (2022). Use of CRISPR/Cas9- based gene editing to simultaneously mutate multiple homologous genes required for pollen development and male fertility in maize. Cells 11 (3), 439. 10.3390/cells11030439 35159251 PMC8834288

[B99] Lloyd A. Plaisier C. L. Carroll D. Drews G. N. (2005). Targeted mutagenesis using zinc- finger nucleases in Arabidopsis. Proc. Natl. Acad. Sci. U. S. A. 102, 2232–2237. 10.1073/pnas.0409339102 15677315 PMC548540

[B100] Lou D. Wang H. Liang G. Yu D. (2017). OsSAPK2 confers abscisic acid sensitivity and tolerance to drought stress in rice. Front. Plant Sci. 8, 993. 10.3389/fpls.2017.00993 28659944 PMC5468418

[B205] Lu Y. Zhu J. K. (2017). Precise editing of a target base in the rice genome using a modified CRISPR/Cas9 system. Mol. Plant 10 (3), 523–525. 27932049 10.1016/j.molp.2016.11.013

[B101] Lu Y. Tian Y. Shen R. Yao Q. Zhong D. Zhang X. (2020). Precise genome modification in tomato using an improved prime editing system. Plant Biotechnol. J. 19, 415–417. 10.1111/pbi.13497 33091225 PMC7955883

[B102] Ma L. Zhang D. Miao Q. Yang J. Xuan Y. Hu Y. (2017). Essential role of sugar transporter *OsSWEET11*during the early stage of rice grain filling. Plant Cell Physiol. 58, 863–873. 10.1093/pcp/pcx040 28371825

[B103] Macovei A. Sevilla N. R. Cantos C. Jonson G. B. Slamet-Loedin I. Čermák T. (2018). Novel alleles of rice eIF4G generated by CRISPR/Cas9-targeted mutagenesis confer resistance to Rice tungro spherical virus. Plant Biotechnol. J. 16 (11), 1918–1927. 10.1111/pbi.12927 29604159 PMC6181218

[B104] Makarova K. S. Wolf Y. I. Alkhnbashi O. S. Costa F. Shah S. A. Saunders S. J. (2015). An updated evolutionary classification of CRISPR–Cas systems. Nat. Rev. Microbiol. 13 (11), 722–736. 10.1038/nrmicro3569 26411297 PMC5426118

[B105] Malabarba J. Chevreau E. Dousset N. Veillet F. Moizan J. Vergne E. (2021). New strategies to overcome present CRISPR/Cas9 limitations in apple and pear: efficient dechimerization and base editing. Int. J. Mol. Sci. 22 (1), 319. 10.3390/ijms22010319 PMC7795782 33396822

[B106] Mao Y. Zhang H. Xu N. Zhang B. Gou F. Zhu J. K. (2013). Application of the CRISPR–Cas system for efficient genome engineering in plants. Mol. Plant 6 (6), 2008–2011. 10.1093/mp/sst121 23963532 PMC3916745

[B107] Martínez M. I. S. Bracuto V. Koseoglou E. Appiano M. Jacobsen E. Visser R. G. (2020). CRISPR/Cas9-targeted mutagenesis of the tomato susceptibility gene PMR4 for resistance against powdery mildew. BMC plant Biol. 20 (1), 1–13. 32560695 10.1186/s12870-020-02497-y PMC7304142

[B108] Miglani G. S. Kaur A. Lovepreet Kaur L. (2020). Plant gene expression control using genome- and epigenome-editing technologies. J. Crop Improv. 34, 1–63. 10.1080/15427528.2019.1678541

[B109] Miglani G. S. Singh R. (2020). “5 epigenome editing in crop improvement,” in Quantitative genetics, genomics and plant breeding. 2nd edition (UK: CAB International), 44–70.

[B110] Modrzejewski D. Hartung F. Lehnert H. Sprink T. Kohl C. Keilwagen J. (2020). Which factors affect the occurrence of off-target effects caused by the use of CRISPR/cas: a systematic review in plants. Front. Plant Sci. 11, 574959. 10.3389/fpls.2020.574959 33329634 PMC7719684

[B111] Mojica M. J. Juez G. Rodríguez-Valera F. (1993). Transcription at different salinities of *Haloferax mediterranei* sequences adjacent to partially modified PstI sites. Mol. Microbiol. 9, 613–621. 10.1111/j.1365-2958.1993.tb01721.x 8412707

[B112] Moore J. K. Haber J. E. (1996). Cell cycle and genetic requirements of two pathways of nonhomologous end-joining repair of double-strand breaks in *Saccharomyces cerevisiae* . Mol. Cell. Biol. 16, 2164–2173. 10.1128/mcb.16.5.2164 8628283 PMC231204

[B113] Nakamura M. Gao Y. Dominguez A. A. Qi L. S. (2021). CRISPR technologies for precise epigenome editing. Nat. Cell Biol. 23, 11–22. 10.1038/s41556-020-00620-7 33420494

[B206] Nishida K. Arazoe T. Yachie N. Banno S. Kakimoto M. Tabata M. (2016). Targeted nucleotide editing using hybrid prokaryotic and vertebrate adaptive immune systems. Science 353 (6305), aaf8729. 27492474 10.1126/science.aaf8729

[B207] Nishimasu H. Shi X. Ishiguro S. Gao L. Hirano S. Okazaki S. (2018). Engineered CRISPR-Cas9 nuclease with expanded targeting space. Science 361 (6408), 1259–1262. 10.1126/science.aas9129 30166441 PMC6368452

[B115] Nuñez J. K. Kranzusch P. J. Noeske J. Wright A. V. Davies C. W. Doudna J. A. (2014). Cas1-Cas2 complex formation mediates spacer acquisition during CRISPR-Cas adaptive immunity. Nat. Struct. Mol. Biol. 21, 528–534. 10.1038/nsmb.2820 24793649 PMC4075942

[B116] Paixão J. F. R. Gillet F. X. Ribeiro T. P. Bournaud C. Lourenço-Tessutti I. T. Noriega D. D. (2019). Improved drought stress tolerance in Arabidopsis by CRISPR/dCas9 fusion with a histone acetyltransferase. Sci. Rep. 9, 8080. 10.1038/s41598-019-44571-y 31147630 PMC6542788

[B117] Papikian A. Liu W. Gallego-Bartolomé J. Jacobsen S. E. (2019). Site-specific manipulation of *Arabidopsis* loci using CRISPR-Cas9 SunTag systems. Nat. Commun. 10, 729. 10.1038/s41467-019-08736-7 30760722 PMC6374409

[B118] Peng A. Chen S. Lei T. Xu L. He Y. Wu L. (2017). Engineering canker-resistant plants through CRISPR/Cas9-targeted editing of the susceptibility gene *CsLOB1* promoter in citrus. Plant Biotechnol. J. 15, 1509–1519. 10.1111/pbi.12733 28371200 PMC5698050

[B119] Perroud P. F. Guyon-Debast A. Veillet F. Kermarrec M. P. Chauvin L. Chauvin J. E. (2022). Prime Editing in the model plant Physcomitrium patens and its potential in the tetraploid potato. Plant Sci. 316, 111162. 10.1016/j.plantsci.2021.111162 35151447

[B120] Puchta H. (2005). The repair of double-strand breaks in plants: mechanisms and consequences for genome evolution. J. Exp. Bot. 56 (409), 1–14. 10.1093/jxb/eri025 15557293

[B121] Pyott D. E. Sheehan E. Molnar A. (2016). Engineering of CRISPR/Cas9-mediated potyvirus resistance in transgene-free Arabidopsis plants. Mol. Plant Pathol. 17 (8), 1276–1288. 10.1111/mpp.12417 27103354 PMC5026172

[B122] Qi L. S. Larson M. H. Gilbert L. A. Doudna J. A. Weissman J. S. Arkin A. P. (2013). Repurposing CRISPR as an RNA-guided platform for sequence-specific control of gene expression. Cell 152, 1173–1183. 10.1016/j.cell.2013.02.022 23452860 PMC3664290

[B123] Qin R. Li J. Li H. Zhang Y. Liu X. Miao Y. (2019). Developing a highly efficient and wildly adaptive CRISPR-SaCas9 toolset for plant genome editing. Plant Biotechnol. J. 17 (4), 706–708. 10.1111/pbi.13047 30537191 PMC6419570

[B124] Quadrana L. Almeida J. Asís R. Duffy T. Dominguez P. G. Bermúdez L. (2014). Natural occurring epialleles determine vitamin E accumulation in tomato fruits. Nat. Commun. 5, 3027. 10.1038/ncomms5027 24967512

[B125] Rahman S. U. McCoy E. Raza G. Ali Z. Mansoor S. Amin I. (2022). Improvement of soybean; A way forward transition from genetic engineering to new plant breeding technologies. Mol. Biotechnol., 1–19. 10.1007/s12033-022-00456-6 35119645

[B126] Rajkumar M. S. Shankar R. Garg R. Jain M. (2020). Bisulphite sequencing reveals dynamic DNA methylation under desiccation and salinity stresses in rice cultivars. Genomics 112, 3537–3548. 10.1016/j.ygeno.2020.04.005 32278023

[B127] Ran F. A. C. L. Cong L. Yan W. X. Scott D. A. Gootenberg J. S. Kriz A. J. (2015). *In vivo* genome editing using *Staphylococcus aureus* Cas9. Nature 520 (7546), 186–191. 10.1038/nature14299 25830891 PMC4393360

[B128] Razzaq A. Saleem F. Kanwal M. Mustafa G. Yousaf S. Imran Arshad H. M. (2019). Modern trends in plant genome editing: an inclusive review of the CRISPR/Cas9 toolbox. Int. J. Mol. Sci. 20, 4045. 10.3390/ijms20164045 PMC6720679 31430902

[B129] Rees H. A. Liu D. R. (2018). Base editing: precision chemistry on the genome and transcriptome of living cells. Nat. Rev. Genet. 19 (12), 770–788. 10.1038/s41576-018-0059-1 30323312 PMC6535181

[B130] Ren B. Yan F. Kuang Y. Li N. Zhang D. Lin H. (2018). A CRISPR/Cas9 toolkit for efficient targeted base editing to induce genetic variations in rice. Sci. China. Life Sci. 60 (5), 516–519. 10.1007/s11427-016-0406-x 28260228

[B208] Rensing S. A. Goffinet B. Meyberg R. Wu S. Z. Bezanilla M. (2020). The moss Physcomitrium (Physcomitrella) patens: A model organism for non-seed plants. Plant Cell 32 (5), 1361–1376. 32152187 10.1105/tpc.19.00828 PMC7203925

[B209] Sanchez-Leon S. Gil-Humanes J. Ozuna C. V. Gimenez M. J. Sousa C. Voytas D. F. (2018). Low-gluten, nontransgenic wheat engineered with CRISPR/Cas9. Plant Biotechnol. J. 16, 902–910. 28921815 10.1111/pbi.12837 PMC5867031

[B131] Sakata R. C. Ishiguro S. Mori H. Tanaka M. Tatsuno K. Ueda H. (2020). Base editors for simultaneous introduction of C-to-T and A-to-G mutations. Nat. Biotechnol. 38 (7), 865–869. 10.1038/s41587-020-0509-0 32483365

[B132] Salomon S. Puchta H. (1998). Capture of genomic and T-DNA sequences during double-strand break repair in somatic plant cells. EMBO J. 17 (20), 6086–6095. 10.1093/emboj/17.20.6086 9774352 PMC1170935

[B133] Semenova E. Jore M. M. Datsenko K. A. Semenova A. Westra E. R. Wanner B. (2011). Interference by clustered regularly interspaced short palindromic repeat (CRISPR) RNA is governed by a seed sequence. Proc. Natl. Acad. Sci. U. S. A. 108, 10098–10103. 10.1073/pnas.1104144108 21646539 PMC3121866

[B134] Shen C. Que Z. Xia Y. Tang N. Li D. He R. (2017). Knock out of the annexin gene OsAnn3 via CRISPR/Cas9-mediated genome editing decreased cold tolerance in rice. J. Plant Biol. 60, 539–547. 10.1007/s12374-016-0400-1

[B135] Shi J. Gao H. Wang H. Lafitte H. R. Archibald R. L. Yang M. (2017). ARGOS8 variants generated by CRISPR-Cas9 improve maize grain yield under field drought stress conditions. Plant Biotechnol. J. 15, 207–216. 10.1111/pbi.12603 27442592 PMC5258859

[B136] Shmakov S. Abudayyeh O. O. Makarova K. S. Wolf Y. I. Gootenberg J. S. Semenova E. (2015). Discovery and functional characterization of diverse class 2 CRISPR-Cas systems. Mol. Cell 60 (3), 385–397. 10.1016/j.molcel.2015.10.008 26593719 PMC4660269

[B137] Shopan J. Lv X. Hu Z. Zhang M. Yang J. (2020). Eukaryotic translation initiation factors shape RNA viruses resistance in plants. Hortic. Plant J. 6 (2), 81–88. 10.1016/j.hpj.2020.03.001

[B138] Shukla V. K. Doyon Y. Miller J. C. DeKelver R. C. Moehle E. A. Worden S. E. (2009). Precise genome modification in the crop species Zea mays using zinc-finger nucleases. Nature 459, 437–441. 10.1038/nature07992 19404259

[B140] Song Q. Zhang T. Stelly D. M. Chen Z. J. (2017). Epigenomic and functional analyses reveal roles of epialleles in the loss of photoperiod sensitivity during domestication of allotetraploid cottons. Genome Biol. 18, 99. 10.1186/s13059-017-1229-8 28558752 PMC5450403

[B141] Song Y. Ji D. Li S. Wang P. Li Q. Xiang F. (2012). The dynamic changes of DNA methylation and histone modifications of salt responsive transcription factor genes in soybean. PLoS One 7, e41274. 10.1371/journal.pone.0041274 22815985 PMC3399865

[B142] Sretenovic S. Liu S. Li G. Cheng Y. Fan T. Xu Y. (2021). Exploring C-To-G base editing in rice, tomato, and poplar. Front. Genome Ed. 24, 756766. 10.3389/fgeed.2021.756766 PMC8525388 34713268

[B143] Sretenovic S. Qi Y. (2022). Plant prime editing goes prime. Nat. Plants 8 (1), 20–22. 10.1038/s41477-021-01047-0 34949803

[B144] Steinert J. Schiml S. Fauser F. Puchta H. (2015). Highly efficient heritable plant genome engineering using Cas9 orthologues from Streptococcus thermophilus and *Staphylococcus aureus* . Plant J. 84 (6), 1295–1305. 10.1111/tpj.13078 26576927

[B145] Streubel J. Blucher C. Landgraf A. Boch J. (2012). TAL effector RVD specificities and efficiencies. Nat. Biotechnol. 30, 593–595. 10.1038/nbt.2304 22781676

[B146] Sun Z. Li N. Huang G. Xu J. Pan Y. Wang Z. (2013). Site-specific gene targeting using transcription activator-like effector (TALE)-based nuclease in *Brassica oleracea* . J. Integr. Plant Biol. 55, 1092–1103. 10.1111/jipb.12091 23870552

[B147] Svitashev S. Schwartz C. Lenderts B. Young J. K. Cigan A. M. (2016). Genome editing in maize directed by CRISPR-Cas9 ribonucleoprotein complexes. Nat. Commun. 7, 13274. 10.1038/ncomms13274 27848933 PMC5116081

[B148] Talakayala A. Ankanagari S. Garladinne M. (2022). CRISPR-cas genome editing system: a versatile tool for developing disease resistant crops. Plant Stress 3, 100056. 10.1016/j.stress.2022.100056

[B149] Tang X. Sretenovic S. Ren Q. Jia X. Li M. Fan T. (2020). Plant prime editors enable precise gene editing in rice cells. Mol. Plant 13, 667–670. 10.1016/j.molp.2020.03.010 32222487

[B150] Tang X. Zheng X. Qi Y. Zhang D. Cheng Y. Tang A. (2016). A single transcript CRISPR-Cas9 system for efficient genome editing in plants. Mol. Plant 9 (7), 1088–1091. 10.1016/j.molp.2016.05.001 27212389

[B151] Tian S. Jiang L. Cui X. Zhang J. Guo S. Li M. (2018). Engineering herbicide- resistant watermelon variety through CRISPR/Cas9-mediated base-editing. Plant Cell Rep. 37 (9), 1353–1356. 10.1007/s00299-018-2299-0 29797048

[B210] Tra M. V. T. Yin X. Bajal I. Balahadia C. P. Quick W. P. Bandyopadhyay A. (2021). “Single base editing using cytidine deaminase to change grain size and seed coat color in rice,” in Rice genome engineering and gene editing. New York, NY: Humana, pp. 135–143. 10.1007/978-1-0716-1068-8_9 33471329

[B152] Ueta R. Abe C. Watanabe T. Sugano S. S. Ishihara R. Ezura H. (2017). Rapid breeding of parthenocarpic tomato plants using CRISPR/Cas9. Sci. Rep. 7, 507. 10.1038/s41598-017-00501-4 28360425 PMC5428692

[B153] Underwood C. J. Choi K. Lambing C. Zhao X. Serra H. Borges F. (2018). Epigenetic activation of meiotic recombination near *Arabidopsis thaliana* centromeres via loss of H3K9me2 and non-CG DNA methylation. Genome Res. 28, 519–531. 10.1101/gr.227116.117 29530927 PMC5880242

[B154] Van Der Oost J. Westra E. R. Jackson R. N. Wiedenheft B. (2014). Unravelling the structural and mechanistic basis of CRISPR–Cas systems. Nat. Rev. Microbiol. 12 (7), 479–492. 10.1038/nrmicro3279 24909109 PMC4225775

[B155] Veillet F. Chauvin L. Kermarrec M. P. Sevestre F. Merrer M. Terret Z. (2019). The Solanum tuberosum GBSSI gene: a target for assessing gene and base editing in tetraploid potato. Plant Cell Rep. 38 (9), 1065–1080. 10.1007/s00299-019-02426-w 31101972

[B156] Veillet F. Kermarrec M. P. Chauvin L. Chauvin J. E. Nogué F. (2020). CRISPR- induced indels and base editing using the *Staphylococcus aureus* Cas9 in potato. PLoS One 15 (8), e0235942. 10.1371/journal.pone.0235942 32804931 PMC7430721

[B157] Wada N. Osakabe K. Osakabe Y. (2022). Expanding the plant genome editing toolbox with recently developed CRISPR-Cas systems. Plant Physiol. 188, 1825–1837. 10.1093/plphys/kiac027 35099553 PMC8968252

[B158] Wang D. Samsulrizal N. H. Yan C. Allcock N. S. Craigon J. Blanco-Ulate B. (2019). Characterization of CRISPR mutants targeting genes modulating pectin degradation in ripening tomato. Plant Physiol. 179 (2), 544–557. 10.1104/pp.18.01187 30459263 PMC6426429

[B159] Wang F. Wang C. Liu P. Lei C. Hao W. Gao Y. (2016). Enhanced rice blast resistance by CRISPR/Cas9-Targeted mutagenesis of the ERF transcription factor gene *OsERF922* . PloS One 11, e0154027. 10.1371/journal.pone.0154027 27116122 PMC4846023

[B160] Wang L. Chen L. Li R. Zhao R. Yang M. Sheng J. (2017). Reduced drought tolerance by CRISPR/Cas9-mediated SlMAPK3 mutagenesis in tomato plants. J. Agric. Food Chem. 65 (39), 8674–8682. 10.1021/acs.jafc.7b02745 28873302

[B161] Wang M. Xu Z. Gosavi G. Ren B. Cao Y. Kuang Y. (2020). Targeted base editing in rice with CRISPR/ScCas9 system. Plant Biotechnol. J. 18 (8), 1645–1647. 10.1111/pbi.13330 31916673 PMC7336372

[B163] Wang W. Ma S. Hu P. Ji Y. Sun F. (2021). Genome editing of rice eIF4G loci confers partial resistance to rice black-streaked dwarf virus. Viruses 13 (10), 2100. 10.3390/v13102100 34696530 PMC8539751

[B164] Wang W. Pan Q. He F. Akhunova A. Chao S. Trick H. (2018). Transgenerational CRISPR-Cas9 activity facilitates multiplex gene editing in allopolyploid wheat. CRISPR J. 1 (1), 65–74. 10.1089/crispr.2017.0010 30627700 PMC6319321

[B165] Wang X. Tu M. Wang D. Liu J. Li Y. Li Z. (2018). CRISPR/Cas9-mediated efficient targeted mutagenesis in grape in the first generation. Plant Biotechnol. J. 16 (4), 844–855. 10.1111/pbi.12832 28905515 PMC5866948

[B166] Wang Y. Cheng X. Shan Q. Zhang Y. Liu J. Gao C. (2014). Simultaneous editing of three homoeoalleles in hexaploid bread wheat confers heritable resistance to powdery mildew. Nat. Biotechnol. 32, 947–951. 10.1038/nbt.2969 25038773

[B167] Wang Z. Liu X. Xie X. Deng L. Zheng H. Pan H. (2021). ABE8e with polycistronic tRNA-gRNA expression cassette significantly improves adenine base editing efficiency in Nicotiana benthamiana. Int. J. Mol. Sci. 22 (11), 5663. 10.3390/ijms22115663 34073486 PMC8198424

[B168] Wu J. Chen C. Xian G. Liu D. Lin L. Yin S. (2020). Engineering herbicide- resistant oilseed rape by CRISPR/Cas9-mediated cytosine base-editing. Plant Biotechnol. J. 1857–1859. 10.1111/pbi.13368 PMC7415781 32096325

[B169] Xie J. Huang X. Wang X. Gou S. Liang Y. Chen F. (2020). ACBE, a new base editor for simultaneous C-to-T and A-to-G substitutions in mammalian systems. BMC Biol. 18, 131. 10.1186/s12915-020-00866-5 32967664 PMC7510086

[B170] Xie K. Minkenberg B. Yang Y. (2015). Boosting CRISPR/Cas9 multiplex editing capability with the endogenous tRNA-processing system. Proc. Natl. Acad. Sci. U. S. A. 112 (11), 3570–3575. 10.1073/pnas.1420294112 25733849 PMC4371917

[B171] Xu R. Kong F. Qin R. Li J. Liu X. Wei P. (2021). Development of an efficient plant dual cytosine and adenine editor. J. Integr. Plant Biol. 63, 1600–1605. 10.1111/jipb.13146 34191398

[B172] Xu R. Li J. Liu X. Shan T. Qin R. Wei P. (2020). Development of plant prime- editing systems for precise genome editing. Plant Commun. 1 (3), 100043. 10.1016/j.xplc.2020.100043 33367239 PMC7747961

[B173] Xu R. Yang Y. Qin R. Li H. Qiu C. Li L. (2016). Rapid improvement of grain weight via highly efficient CRISPR/Cas9-mediated multiplex genome editing in rice. J. Genet. Genomics. 43, 529–532. 10.1016/j.jgg.2016.07.003 27543262

[B174] Xu Y. Lin Q. Li X. Wang F. Chen Z. Wang J. (2021). Fine-tuning the amylose content of rice by precise base editing of the Wx gene. Plant Biotechnol. J. 19 (1), 11–13. 10.1111/pbi.13433 32558105 PMC7769246

[B211] Xu W. Yang Y. Yang B. Krueger C. J. Xiao Q. Zhao S. (2022). A design optimized prime editor with expanded scope and capability in plants. Nat. Plants 8 (1), 45–52. 34949802 10.1038/s41477-021-01043-4

[B175] Yin X. Biswal A. K. Dionora J. Perdigon K. M. Balahadia C. P. Mazumdar S. (2017). CRISPR-Cas9 and CRISPR-Cpf1 mediated targeting of a stomatal developmental gene EPFL9 in rice. Plant Cell Rep. 36 (5), 745–757. 10.1007/s00299-017-2118-z 28349358

[B212] Yuste-Lisbona F. J. Fernández-Lozano A. Pineda B. Bretones S. Ortíz-Atienza A. García-Sogo B. (2020). ENO regulates tomato fruit size through the floral meristem development network. Proc. Natl. Acad. Sci. U S A. 117 (14), 8187–8195. 10.1073/pnas.1913688117 32179669 PMC7148573

[B176] Zeng Y. Wen J. Zhao W. Wang Q. Huang W. (2020). Rational improvement of rice yield and cold tolerance by editing the three genes OsPIN5b, GS3, and OsMYB30 with the CRISPR–Cas9 system. Front. Plant Sci. 10, 1663. 10.3389/fpls.2019.01663 31993066 PMC6964726

[B177] Zetsche B. Gootenberg J. S. Abudayyeh O. O. Slaymaker I. M. Makarova K. S. Essletzbichler P. (2015). Cpf1 is a single RNA-guided endonuclease of a class 2 CRISPR-Cas system. Cell 163 (3), 759–771. 10.1016/j.cell.2015.09.038 26422227 PMC4638220

[B178] Zhang A. Liu Y. Wang F. Li T. Chen Z. Kong D. (2019). Enhanced rice salinity tolerance via CRISPR/Cas9-targeted mutagenesis of the OsRR22 gene. Mol. Breed. 39 (3), 47. 10.1007/s11032-019-0954-y 32803201 PMC7413041

[B179] Zhang C. Wang F. Zhao S. Kang G. Song J. Li L. (2020). Highly efficient CRISPR-SaKKH tools for plant multiplex cytosine base editing. Crop J. 8 (3), 418–423. 10.1016/j.cj.2020.03.002

[B180] Zhang J. Zhang X. Chen R. Yang L. Fan K. Liu Y. (2020). Generation of transgene-free Semidwarf maize plants by gene editing of gibberellin-oxidase20-3 using CRISPR/Cas9. Front. Plant Sci. 104, 1048. 10.3389/fpls.2020.01048 PMC7365143 32742269

[B181] Zhang R. Liu J. Chai Z. Chen S. Bai Y. Zong Y. (2019). Generation of herbicide tolerance traits and a new selectable marker in wheat using base editing. Nat. Plants 5 (5), 480–485. 10.1038/s41477-019-0405-0 30988404

[B182] Zhang X. Zhu B. Chen L. Xie L. Yu W. Wang Y. (2020). Dual base editor catalyzes both cytosine and adenine base conversions in human cells. Nat. Biotechnol. 38 (7), 856–860. 10.1038/s41587-020-0527-y 32483363

[B183] Zhang Y. Bai Y. Wu G. Zou S. Chen Y. Gao C. (2017). Simultaneous modification of three homoeologs of *TaEDR1* by genome editing enhances powdery mildew resistance in wheat. Plant J. 91, 714–724. 10.1111/tpj.13599 28502081

[B184] Zhang Y. Liang Z. Zong Y. Wang Y. Liu J. Chen K. (2016). Efficient and transgene-free genome editing in wheat through transient expression of CRISPR/Cas9 DNA or RNA. Nat. Commun. 7, 12617. 10.1038/ncomms12617 27558837 PMC5007326

[B185] Zhao C. Zhang Z. Xie S. Si T. Li Y. Zhu J. K. (2016). Mutational evidence for the critical role of CBF genes in cold acclimation in *Arabidopsis* . Plant Physiol. 171, 2744–2759. 10.1104/pp.16.00533 27252305 PMC4972280

[B186] Zhao D. Li J. Li S. Xin X. Hu M. Price M. A. (2021). Glycosylase base editors enable C-to-A and C-to-G base changes. Nat. Biotechnol. 39 (1), 35–40. 10.1038/s41587-020-0592-2 32690970

[B188] Zhong X. Du J. Hale C. J. Gallego-Bartolome J. Feng S. Vashisht A. A. (2014). Molecular mechanism of action of plant DRM de novo DNA methyltransferases. Cell 157, 1050–1060. 10.1016/j.cell.2014.03.056 24855943 PMC4123750

[B213] Zhong Z. Sretenovic S. Ren Q. Yang L. Bao Y. Qi C. (2019). Improving plant genome editing with high-fidelity xCas9 and non-canonical PAM-targeting Cas9-NG. Mol. Plant 12 (7), 1027–1036. 30928637 10.1016/j.molp.2019.03.011

[B189] Zhou J. Peng Z. Long J. Sosso D. Liu B. Eom J. S. (2015). Gene targeting by the TAL effector PthXo2 reveals cryptic resistance gene for bacterial blight of rice. Plant J. 82, 632–643. 10.1111/tpj.12838 25824104

[B190] Zhou J. Xin X. He Y. Chen H. Li Q. Tang X. (2019). Multiplex QTL editing of grain-related genes improves yield in elite rice varieties. Plant Cell Rep. 38 (4), 475–485. 10.1007/s00299-018-2340-3 30159598

[B214] Zhuang Y. Liu J. Wu H. Zhu Q. Yan Y. Meng H. (2022). Increasing the efficiency and precision of prime editing with guide RNA pairs. Nat. Chem. Biol. 18 (1), 29–37. 34711981 10.1038/s41589-021-00889-1

[B191] Zong Y. Wang Y. Li C. Zhang R. Chen K. Ran Y. (2017). Precise base editing in rice, wheat and maize with a Cas9-cytidine deaminase fusion. Nat. Biotechnol. 35 (5), 438–440. 10.1038/nbt.3811 28244994

[B215] Zsogon A. Cermak T. Naves E. R. Notini M. M. Edel K. H. Weinl S. (2018). De novo domestication of wild tomato using genome editing. Nat. Biotechnol. 36, 1211–1216. 10.1038/nbt.4272 30272678

